# Expression Patterns of Histone Modification Regulators Predict Prognosis and Microenvironment Characteristics in Gastric Cancer

**DOI:** 10.1049/syb2.70089

**Published:** 2026-11-04

**Authors:** Yue Huang, Ya Gao, Fuli Xie, Xiaoxiang Rong, Rui Zhou, Zhenhua Huang, Kuncai Liu, Jiayun Guan, Zewei Li, Siting Zheng, Jianhua Wu, Guangting He, Yanhua Ou, Huiying Sun

**Affiliations:** ^1^ Department of Oncology Nanfang Hospital Southern Medical University Guangzhou China; ^2^ Affiliated Qingyuan Hospital Guangzhou Medical University Qingyuan People's Hospital Qingyuan Guangdong China; ^3^ Department of Anesthesiology Nanfang Hospital Southern Medical University Guangzhou China; ^4^ Medical Education Center Nanfang Hospital Southern Medical University Guangzhou China

**Keywords:** biocomputing, gastric cancer, histone modification, patient prognosis, pattern classification, tumour microenvironment

## Abstract

Multiple studies have demonstrated a significant correlation between histone modifications and tumour initiation and progression. However, it remains unclear whether the histone modification profile contributes to the classification of patients with gastric cancer (GC). After analysing transcriptomic data from 76 histone modification regulators (HMR), we identified four distinct patterns in 838 GC specimens. These patterns demonstrated associations with different prognoses, treatment responses, biological pathways, and tumour microenvironment characteristics. Therefore, we used the Least Absolute Shrinkage and Selection Operator (LASSO) regression and multivariate Cox regression analysis to explore GC's histone modification regulator characteristics and established a 10‐gene scoring scheme named “Histone Modification Regulator‐based Risk Score (HR_score)” to cluster tumours into high‐ and low‐risk subtypes. Patients with high scores are characterised by increased stromal cell infiltration and exhibit the activation of innate immunity. These observations may be linked to the immune‐exclusion phenotype and a poor prognosis. Further analysis revealed a direct correlation between higher HR_score and diminished response to adjuvant chemotherapy and immunotherapy treatments. In summary, this study indicates that HR_score can serve as a robust prognostic tool for GC, which can effectively perform patient risk stratification and predict treatment response, providing potential value for guiding individualised treatment strategies.

## Graphical abstract

We used the Least Absolute Shrinkage and Selection Operator (LASSO) regression and multivariate Cox regression analysis to explore GC's histone modification regulator characteristics and established a 10‐gene scoring scheme named “HR_score” to clustered tumours into low, high subtypes. This study demonstrated that the HR_score could be instrumental in predicting the prognosis of GC patients, and assessing the effectiveness of postoperative adjuvant chemotherapy.

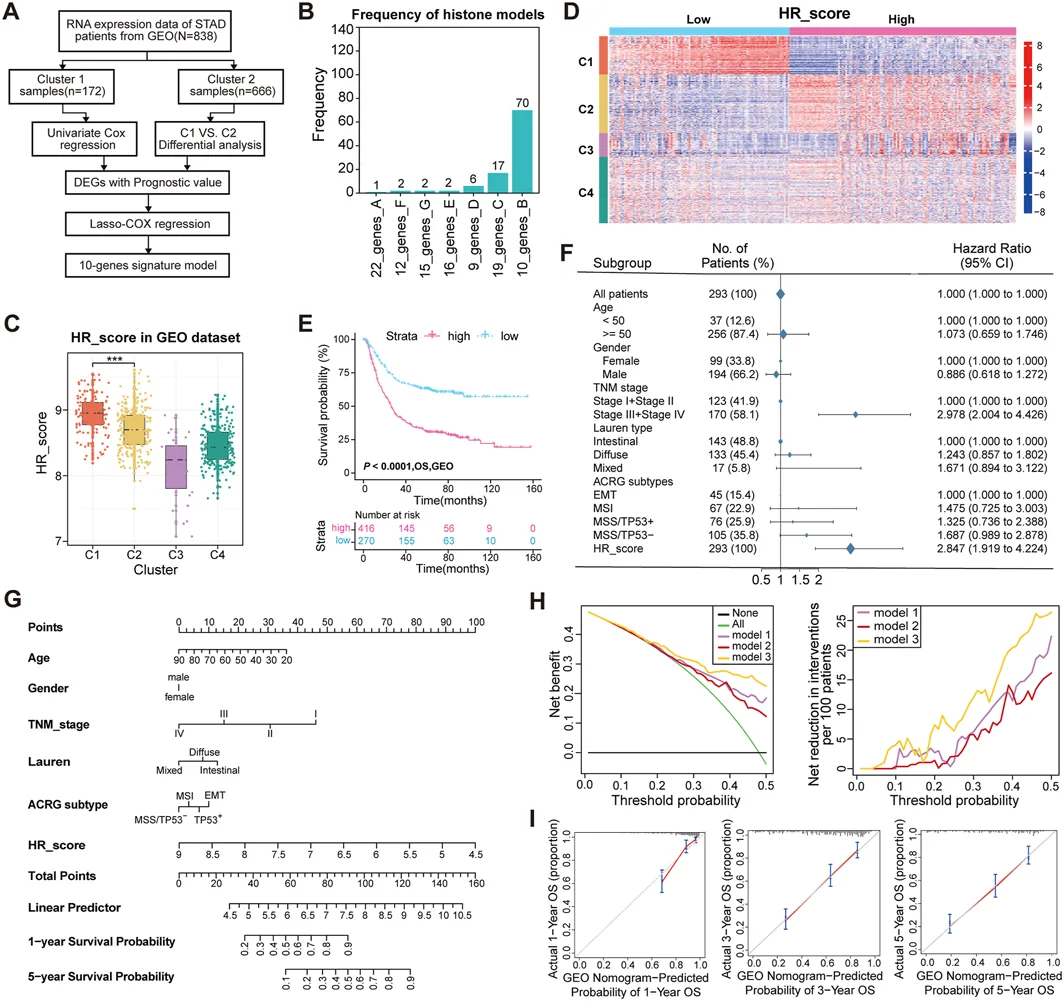

**Abbreviations**
AJCC: American joint committee on cancer
APM: antigen processing and presenting machinery
AUC: area under the curve
CI: confidence interval
CMap: connectivity map
CNV: copy number variation
CTL: cytotoxic T lymphocyte
DC: dendritic cell
DCA: decision curve analysis
DEGs: differentially expressed genes
DMS: DNA methylation score
EMT: epithelial‐mesenchymal transition
GC: gastric cancer
GEO: gene expression synthesis
GSVA: gene set variant analysis
H3K4: H3 lysine 4
HAT: histone acetyltransferase
HDAC: histone deacetylase
HMR: HMR‐C1 histone modification regulator histone modification regulator cluster 1
HR: hazard ratio
HR_score: histone modification regulator risk score
ICIs: immune checkpoint inhibitors
LASSO: least absolute shrinkage and selection operator
OS: overall survival
Pan‐F‐TBRs: pan‐fibroblast TGF‐β response
RMA: PFS robust multi‐array average progression‐free survival
ssGSEA: single‐sample gene set enrichment analysis
TCGA: the cancer genome atlas
TIDE: tumour immune dysfunction and exclusion
TME: tumour microenvironment
YAP: yes‐associated protein

## 1 Introduction

Gastric cancer (GC) is the fifth most common malignancy and the third leading cause of cancer‐related deaths in China [1]. Currently, the TNM staging system is the most widely used clinical staging system for GC. However, the TNM staging system lacks the molecular characteristics of patients, and patients at the same stage often show significant differences in prognosis and response to different treatment strategies. Therefore, developing new indicators based on patient molecular characteristics could aid in the accurate diagnosis and treatment of GC. In recent years, several molecular typing schemes have been proposed, such as The Cancer Genome Atlas (TCGA) genotyping and ACRG typing. However, the high‐throughput technology used in these typing methods are expensive and complex. Hence, there is a need for the development of economical, convenient, and easy clinical transformation methods for molecular typing.

Currently, epigenetic alterations are emerging as clinical biomarkers for tumour‐related diagnosis, prognosis, and treatment [2]. In the field of GC, several scoring models based on epigenetic modifications have been proposed. For instance, Qi Meng et al. [3] construct a DNA methylation score (DMS) aimed at enhancing the identification of the immune microenvironment in GC. Similarly, Bo Zhang et al. [4] developed an m6A score by analysing m6A modification patterns and linking them to tumour microenvironment (TME) cell infiltration characteristics. However, as another important type of epigenetic regulation, histone modification plays a crucial role in tumour *o* initiation and progression by affecting chromatin structure and transcription factor accessibility. Compared with DNA methylation, histone modification exhibits stronger dynamic reversibility, and its regulators often serve as drug targets, offering greater potential for clinical translation.

As an important epigenetic modification, dysregulation of histone modifications can lead to aberrant transcriptional programs, thereby affecting tumour immunogenicity and the function of immune cells involved in the antitumour response. Alterations in histone acetylation or methylation patterns and their interactions have been identified as highly impacted epigenetic pathways in tumours [5, 6, 7]. Histone methylation is one of the most important post‐translational modifications, primarily involving the addition of methyl groups to lysine residues of histones H3 and H4 [8]. This modification can serve as either an activating or repressive marker for gene expression. On the other hand, histone acetylation is primarily regulated by the balance between histone acetyltransferases (HAT) and histone deacetylases (HDAC). Acetylation of lysine residues on the histone tails reduces the positive charge of histones, decreasing their electrostatic affinity for DNA, which results in the relaxation of the chromatin structure, making the open chromatin conformation more accessible to transcription factors and significantly increasing gene expression [9]. Therefore, histone acetylation is generally considered an active histone marker [10]. Acetylation and methylation of the same lysine residue exhibit a competitive relationship, as the two modifications typically do not occur simultaneously, thereby allowing different types of histone modifications to influence one another [11].

Recently, aberrant expression of histone modification enzymes and their various catalytic modifications has been identified in the initiation, progression, and metastasis of GC [12, 13]. Abnormal histone modification pattern may serve as a driving factor in GC. However, despite the clinical significance of histone modifications in GC, their direct detection and validation in large‐scale clinical cohorts remain challenging. High technical requirements, substantial costs, and the need for high‐quality fresh tissue limit their widespread application in routine clinical diagnostics. To bridge this gap, the emerging field of computational oncology has become a powerful tool for deciphering complex biological patterns [14, 15]. Advanced computational frameworks have been successfully applied to integrate multi‐omics data, identify robust molecular subtypes, and characterise the TME in GC [16, 17, 18]. Furthermore, the integration of diverse information sources through artificial intelligence facilitates the discovery of latent epigenetic features that traditional methods might overlook [19]. However, due to previous technical limitations, most studies have focused on only a few histone modification regulators. Given the highly integrated interactions among these modifications, exploring the regulatory network of tumour heterogeneity and understanding the biological functions of the TME may contribute to the development of individualised treatment strategies for GC.

Therefore, this study systematically evaluated the expression patterns of 76 histone acetylation and methylation regulators in gastric cancer, and explored their relationships with patient prognosis, tumour microenvironment characteristics, and treatment response. We put forward the following research hypothesis: the expression patterns of histone modification regulators can be used for molecular typing of gastric cancer patients, and the prognostic scoring model constructed based on this can effectively predict patient prognosis and guide individualised treatment. To achieve this goal, we first identified different subtypes of histone modification regulators using multi‐centre gastric cancer cohort data, then constructed and validated a quantitative scoring model named Histone modification regulator Risk score (HR_score), and finally evaluated the clinical value of this model in prognosis prediction and treatment guidance.

## 2 Methods

### 2.1 Collection and Pretreatment of the Gastric Cancer Dataset

Public transcriptomic and clinical data of gastric cancer samples were retrospectively retrieved from the NCBI GEO database (https://www.ncbi.nlm.nih.gov/geo/), including the GSE62254/ACRG cohort (*n* = 300), GSE57303 (*n* = 70), GSE29272 (*n* = 268), GSE15459 (*n* = 200). These datasets were consolidated as the meta‐GEO cohort (*n* = 838) and used as a training set for the gastric cancer risk assessment model (Table S1). Gene name conversion was performed for probe names based on platform annotation files such as GPL570, GPL96, etc., and if multiple probes corresponding to the same genes existed, the maximum value of multiple probes in these genes was retained. Raw data for the Affymetrix dataset were background adjusted, quantile normalised using the RMA algorithm in the Affy package, and a final summary of oligonucleotides for each transcript was made using the median optimisation algorithm. Corresponding clinical data were obtained from the supplementary files of the relevant articles. To verify the effectiveness of batch effect correction, we used principal component analysis (PCA) to visually compare the sample distribution before and after correction. Before correction, samples from different GEO cohorts showed obvious separation; after correction using the sva‐ComBat function in the “SVA” R package, the distribution of samples from each cohort tended to be mixed, indicating that the batch effect was effectively controlled (Supporting Information S1: Figure S1).

Gene expression data of gastric cancer (TCGA‐STAD) were downloaded from the UCSC Xena database (https://xenabrowser.net/), and the Homo_sapiens.GRCh38.99.chr.gtf genome annotation file was used for gene ID transformation and normalised the data as a validation set for the gastric cancer risk assessment model. Up‐to‐date clinical data, sample information, somatic mutation data, and DNA methylation data for TCGA‐STAD samples were obtained from the database (https://portal.gdc.cancer.gov/) using the TCGAbiolinks R package. Of these, somatic mutation data were analysed using maftools to compare the characteristics of the distribution of somatic mutations in the high HR_score and low HR_score groups.

For external validation, RNA sequencing data from the TIMES00l cohort (Nanfang Hospital, Southern Medical University) were utilised [20]. This cohort comprised 86 pre‐treatment advanced gastric cancer tissue samples, and the original bulk RNA‐seq data have been deposited at GSA‐Human (https://ngdc.cncb.ac.cn/gsa‐human/, accession number: HRA005161).

### 2.2 Gene Set Variant Analysis (GSVA)

The expression matrix of genes across different samples was transformed into the expression matrix of gene sets across samples and analysed using the GSVA algorithm of the R package “GSVA” to download the “Hallmark” gene sets from the MSigDB database.

### 2.3 Characterization of the Tumour Microenvironment

The Tumour Immune Dysfunction and Exclusion (TIDE) algorithm (http://tide.dfci.harvard.edu/) predicts treatment response to immune checkpoint blockade between groups. This is a computational method that simulates various tumour immune escape: including tumour‐infiltrating cytotoxic T lymphocyte (CTL) dysfunction and immunosuppressive CTL exclusion [21]. The results are also considered to be able to replace a single biomarker to effectively predict the effect of immune checkpoint inhibition therapy. A higher TIDE score indicates a greater likelihood of tumour cells inducing immune escape and a lower response rate to treatment with immune checkpoint inhibitors (ICIs).

The relative abundance of each immune cell infiltrate in the GC TME was estimated using the single‐sample gene set enrichment analysis (ssGSEA) algorithm in the R package “GSVA”. The relative abundance of each TME infiltrating cell is indicated by the enrichment score in the ssGSEA analysis, and gene sets for each immune cell subset were obtained from Bindea et al. [22]. Based on the transcriptome expression data using the ESTIMATE algorithm to predict the levels of immune cells and stromal cells in malignant tumours, generate ESTIMATE score to evaluate tumour purity, the lower the sample purity, the more stromal components. Using the I OBR package developed by Zeng et al. [23], Antigen processing and presentation mechanism (antigen processing and presenting machine, APM) scores, Pan‐fibroblast TGF‐β response (Pan‐F‐TBRS) scores of high‐ and low‐score groups were calculated using the ssGSEA method.

### 2.4 Construction of the Prognostic Model

In order to establish a clinical risk assessment model for different populations of gastric cancer patients, GEO data (GSE62254, GSE57303, GSE29272, and GSE15459) were used as training set and the TCGA‐STAD cohort as validation set. To screen for genes related to histone modification regulators, the limma package was used to perform differential expression analysis between histone modification regulator cluster 1 and non‐histone modification regulator cluster 1 subtypes in the meta‐GEO cohort, with the screening thresholds set as *p* < 0.001 and |log2FC| ≥ 1. Subsequently, the Boruta algorithm was employed to reduce the dimensionality of differentially expressed genes (DEGs) and screen for core feature genes (parameter settings: doTrace = 2, maxRuns = 100, ntree = 500). Then, univariate Cox regression analysis was conducted to screen for genes with significant prognostic value (*p* < 0.05). To construct the optimal prognostic model, LASSO‐COX regression analysis was performed using the glmnet package, with ten‐fold cross‐validation adopted to select the *λ* value corresponding to the minimum partial likelihood deviance as the optimal regularisation parameter. Finally, 10 genes were screened out to construct a multivariate Cox regression clinical prediction model. The risk score (HR_score) for each patient was defined as a weighted linear combination of the expression values of each gene, that is,

HR_score=β1×ACVR1+β2×BRD3+…+β10×INFA2,

where βi is the regression coefficient of each gene in the multivariate Cox model. The regression coefficients were estimated using the training set (meta‐GEO cohort) and applied to the validation sets (TCGA‐STAD and TIMES001 cohorts) (Table S2).

### 2.5 Construction and Validation of the Nomograms

Multivariate Cox regression analysis was performed to determine the histone modification model scores independent of the screened clinical characteristics. Nomograms were then constructed based on the multivariate Cox regression coefficients of regulator modification characteristics and clinical characteristics for histones in the GSE62254 cohort and the TCGA‐STAD cohort, and calibration curves were plotted to check the agreement between the predicted 1‐, 3‐, and 5‐year Overall Survival (OS) probabilities and the actual observed values.

### 2.6 Correlation Analysis of Drug Sensitivity and Potential Drug Selection

Using the CTRP v2 database [24, 25] (https://portals.broadinstitute.org/ctrp.v2.1/) and PRISM, a molecular barcoding method [26], we predicted the sensitivity of HR _score to chemotherapeutic agents. The CTRP v2 database contains sensitivity data for 481 compounds, multiple omics data on mutation, gene expression, and copy number variation in 860 cancer cell lines, and PRISM contains sensitivity data for 1448 compounds. The area under the curve (AUC) calculated by PRISM and CTRP v2 were used as measures of sensitivity to predict the potential therapeutic agents in histone modification regulators (HMR) typing and high and low HR_score groups, resulting in 4 CTRP database sources and 9 PRISM sources. Using the Connectivity map (CMap) approach, a database of gene expression profiles based on the expression of intervening genes developed by the Broad Institute and containing gene expression profiles generated by treating cells with 1309 known functional agents, the molecular characteristics of gastric cancer were obtained by differential analysis of tumour and normal samples in the high‐ and low‐score groups in the TCGA‐STAD cohort, and CMap analysis was used to calculate the CMAP score, with higher negative connectivity scores indicating that the drug may have potential therapeutic value due to its potential to reverse the molecular signature of gastric cancer.

### 2.7 The CNV Mutation Profile and its Methylation Characteristics

Somatic mutation data were downloaded using the “TCGAbiolinks” package, and differences in somatic mutation distribution between high and low HR_score groups in the TCGA‐STAD cohort were visualised using the “maftools” package. Next, TCGA‐STAD methylation data were obtained from UCSC Xena (https://xenabrowser.net), and GISTIC_2.0 online analysis based on CNV alterations was calculated through the GenePattern website (https://cloud.genepattern.org/) to obtain GISTIC scores for the high and low HR_score subgroups. The GISTIC2 analysis can detect genomic regions that are significantly amplified or missing in the samples, and information on significantly amplified and missing regions in this batch is calculated by analysing the CNV assay results for each sample.

### 2.8 Cell Culture

Human gastric cancer cell lines AGS and MKN45 and the normal gastric epithelial cell line GES‐1 were purchased from Procell Life Science & Technology Co. Ltd, China. AGS cells were cultured in Ham's F‐12K medium (Cat #PM150910, Procell), MKN45 cells in RPMI‐1640 medium (Cat #PM150110, Procell), and GES‐1 cells in DMEM high‐glucose medium (Cat #PM150210, Procell). All media were supplemented with 10% foetal bovine serum (FBS, Cat #164210‐50, Procell) and 1% penicillin‐streptomycin (Cat #15140122, Gibco). Cells were maintained at 37°C in a humidified atmosphere containing 5% CO_2_. When cells reached 80%–90% confluence, they were passaged using 0.25% trypsin‐EDTA (Cat #C100C1, NCM Biotech).

### 2.9 Quantitative Real‐Time PCR (qRT‐PCR)

Total RNA was extracted from cells using Trizol reagent (Cat #15596026, Invitrogen) according to the manufacturer's instructions. Subsequent steps were performed as previously described [27]. Each reaction was performed in triplicate. The primer sequences (Table S3) and *β‐ACTIN* (internal control) were used. Relative mRNA expression levels were calculated using the 2^–ΔΔCt method, with GES‐1 cells as the reference. Data are presented as mean ± standard deviation from three independent experiments. Statistical comparisons between groups were performed using Student's t‐test, with *p* < 0.05 considered statistically significant.

### 2.10 Statistical Analysis

Data were analysed using R software version 4.1.3, the R Bioconductor package and version IBM SPSS Statistics 26.0. All statistical tests were two‐tailed. Student's t‐test (for normally distributed data) or Mann‐Whitney *U* test (for non‐normally distributed data) was used for comparisons between two groups; one‐way analysis of variance or Kruskal‐Wallis test was employed for comparisons among multiple groups as appropriate. Fisher's exact test or Pearson's chi‐square test was used for comparisons of categorical variables. Pearson or Spearman correlation analysis was conducted for correlation analysis. Survival curves were estimated using the Kaplan‐Meier method, and log‐rank test was used for comparisons between groups. Univariate and multivariate Cox proportional hazards regression models were adopted to screen for prognostic factors. To control the false positive rate in multiple comparisons, the Benjamini‐Hochberg method was used to adjust *p* values in the following analyses, with an adjusted *p* value (FDR) < 0.05 considered statistically significant: differential expression analysis (limma), univariate Cox regression screening, and Boruta feature selection. Unless otherwise stated, statistical significance was set at *p* < 0.05. The significance markers in the figures are as follows: ns, not significant; *p < 0.05; **p < 0.01; ***p < 0.001; ****p < 0.0001.

## 3 Results

### 3.1 Transcriptomics and Genetic Variation Landscape of Histone Regulators in GC

Based on a review of histone acetylation and methylation, we have summarised 76 known putative regulators of histone modifications. These regulators include 14 acetyltransferases, 16 deacetyl transferases, 26 methyltransferases, and 20 demethylases, which will be further analysed (Supporting Information S2: Table S4‐1). To investigate the characteristics of histone modification regulators in patients with GC, we collected gene expression profiles from 350 GC samples and 57 non‐tumour normal tissue samples from the TCGA‐STAD database, and analysed the expression levels and prognostic significance of these histone regulators in tumours (Figure 1A–D). The results showed that the expression of most regulators differed significantly between tumour and normal tissues. Compared with normal tissues, more histone regulators were highly expressed in tumour tissues (e.g., KMT2B, KMT5B, NCOA1, HDAC5, etc.), and the expression of some regulators was significantly associated with patient overall survival (OS) (Supporting Information S2: Table S4‐2). These findings indicate that histone modifications play important roles in the development and progression of GC.

**FIGURE 1:**
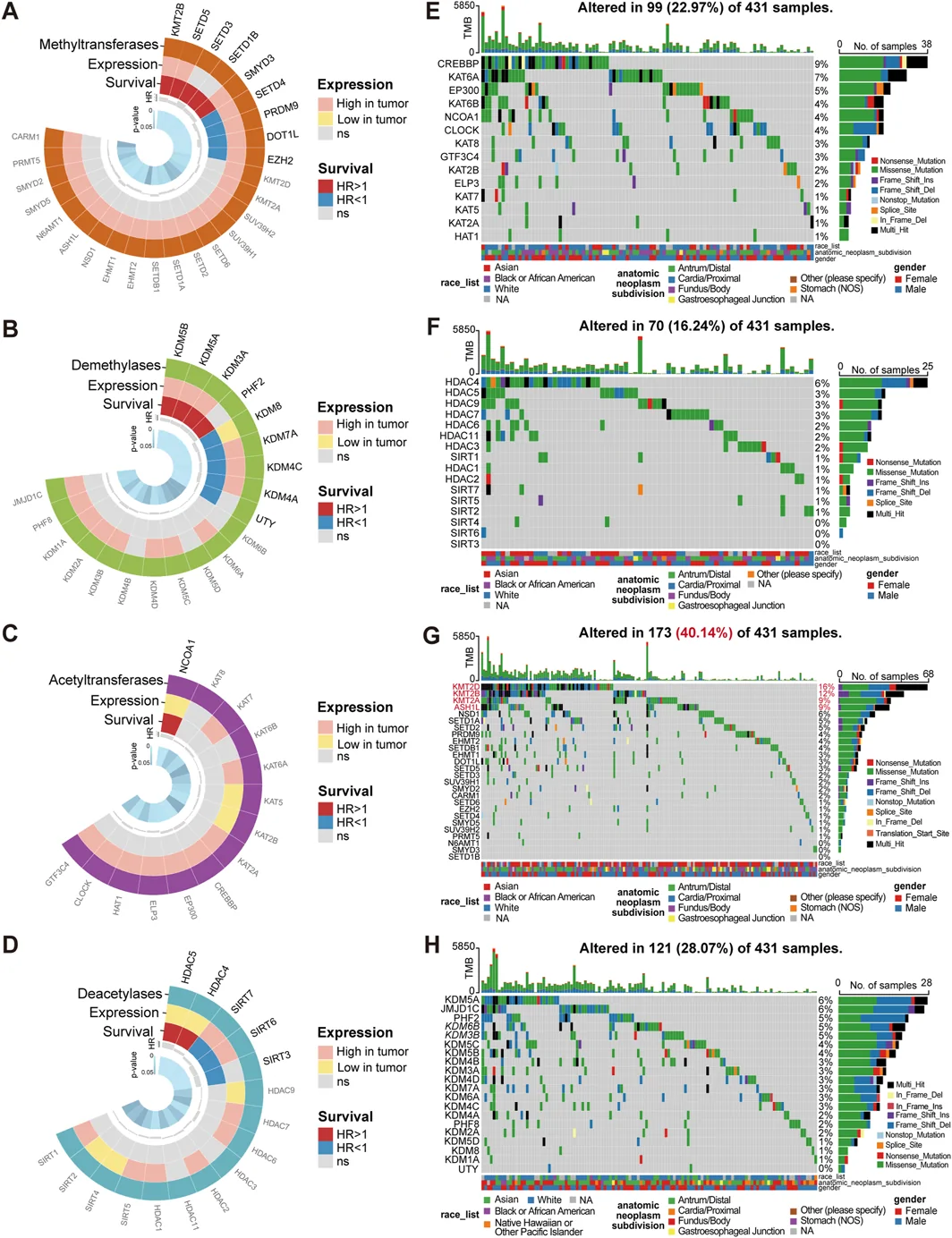
Transcriptomic and genetic alterations of histone modification regulators in gastric cancer. (A–D). The Circo diagram represents the expression and prognosis of histone methyltransferase (A), histone demethylase (B), histone acetyltransferase (C), and histone deacetylase (D) regulators in patients with gastric cancer. The outer circle (A–D) is brown, green, purple, and blue for histone methyltransferase (A), histone demethylase (B), histone acetyltransferase (C), and histone deacetylase (D) respectively. Explanatory Note for Circular Plots: The outer colour bar identifies the regulator category. In the inner tracks, pink represents upregulation of genes in gastric cancer tissues relative to normal tissues, and yellow represents downregulation. Blue in the circle represents favourable factors for overall survival (HR > 1, *p* < 0.05), red represents the risk factor for overall survival (HR < 1, *p* < 0.05), and grey represents factors that have no statistically significant effect on overall survival (*p* > 0.05). The inner circle represents the *p*‐value of the HR values. (E–H). The waterfall plot represents the frequency of mutations in histone methyltransferase (E), histone demethylase (F), histone acetyltransferase (G), and histone deacetylase (H), in the TCGA‐STAD cohort. Each column in the figure represents one patient. The top bars represent the total tumour mutation burden of the patient. Numbers on the right show the mutation frequency of each regulator. The bar graph on the right shows the proportion of each variant type.

Next, we analysed the mutation frequency of these regulators in somatic cells (Figure 1E–H). Analysis of Somatic mutation data from 431 samples in the TCGA‐STAD database revealed that acetyltransferase genes had the highest mutation frequency (40.14%) among the 431 samples included, and the four histone modification regulators with the highest mutation frequency in GC were KMT2D (69/431, 16%), KMT2B (52/431, 12%), KMT2A (39/431, 9%), and ASH1L (39/431, 9%) (Figure 1G). Notably, all of these regulators are histone H3 lysine 4 (H3K4) methyltransferases (Figure 1E–H) and belong to the “Complex of proteins associated with Set1 (COMPASS)” family [28]. Previous studies have demonstrated that SET1A promotes the development and progression of breast, lung, and colorectal cancer through the methylation of histone and non‐histone substrate YAP [29]. However, most of the regulators have a low mutation rate of less than 10%. In conclusion, the differential gene expression and somatic mutation frequencies of histone modification regulators suggest that their unbalanced expression may play a key role in the development and progression of GC.

### 3.2 Identification of the Expression Patterns of the Histone Regulators

It has been reported that histone modifications play a crucial role in the initiation and progression of GC through epigenomic reprogramming [30]. To assess whether transcriptional analysis of 76 acetylation and methylation regulators contributes to the classification of GC patients, we included a total of 838 patients with stage I‐IV GC from four independent GC cohorts (GSE15459, GSE29272, GSE57303, and GSE62254) (Supporting Information S2: Table 3‐S4). Unsupervised K‐means cluster analysis was performed on these four GEO cohorts (meta‐GEO cohort, 838 cases) to identify distinct histone modification expression patterns in GC. The optimal number of clusters was selected using the R package “ConsensusClusterPlus”, resulting in four distinct expression patterns of histone modification regulators, which were named Histone modification regulator cluster 1 (HMR‐C1) (*n* = 172), HMR‐C2 (*n* = 262), HMR‐C3 (*n* = 107), and HMR‐C4 (*n* = 297) (Supporting Information S1: Figure S2). We analysed the differential expression of histone modification regulators across the four HMR subtypes in the meta‐GEO cohort (Figure 2A). Most histone modification regulators were enriched in HMR‐C1, HMR‐C2, and HMR‐C3, indicating strong activity of histone modifications in these three clusters. Among them, *SETD5*, *SETD3*, *SETD1B*, *PHF2*, *NCOA1*, *HDAC5*, and *HDAC4* were relatively highly expressed in HMR‐C1. We hypothesised that these methylation or acetylation regulators might play highly integrated interactions in tumour biological processes and TME, and ultimately participate in the development of heterogeneity of gastric cancers and influence prognosis. Analysis of the clinical characteristics of the four HMR subtypes showed no significant differences in sex, age, or TNM staging (Supporting Information S2: Table 4‐S4; Figure 2B). Survival analysis of the meta‐GEO cohort showed that the OS time of HMR‐C1 was significantly shorter than the other three subtypes (HMR‐C1 vs. HMR‐C2,3,4) with a hazard ratio (HR) of 2.419, 95% confidence interval (CI) of 1.684–3.475 (Figure 2C). Multivariate COX regression analysis based on the clinical characteristics of patients in the GSE62254 cohort showed that HMR‐C1 was an independent poor prognostic factor, with an HR of 2.743 and a 95% CI of 1.820–4.135 (Figure 2D).

**FIGURE 2:**
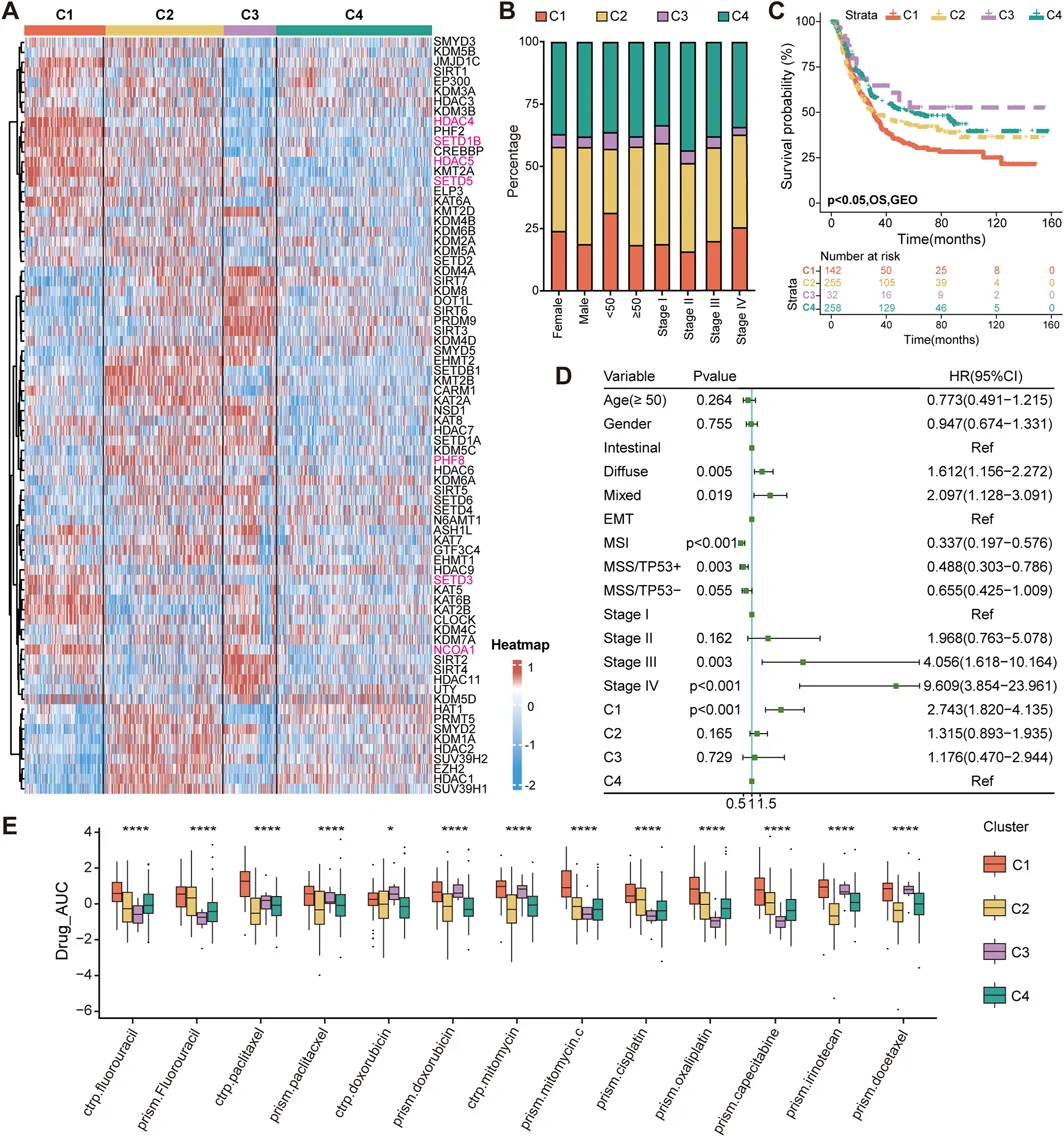
Consensus clustering of histone modification regulators in gastric cancer patients. (A) Unsupervised cluster analysis of 76 histone regulatory factors in the meta‐GEO cohort identified a heat map of histone regulatory factor expression patterns in gastric cancer patients. (B) Bars represent the percentage of HMR typing in different clinical information subgroups. (C) Kaplan‐Meier curves of OS were plotted based on histone modification regulatory factor patterns in the meta‐GEO cohort. (D) Forest plot demonstrating multifactor Cox regression analysis based on clinical characteristics of patients in the GSE62245 cohort. Unadjusted HR (box) and 95% confidence intervals (horizontal line) are depicted. (E) HMR typing based on predicted sensitivities to commonly used adjuvant chemotherapeutic agents from the CTRPv2 and PRISM databases. Boxes represent interquartile ranges (25%–75% data range), horizontal lines in boxes represent medians, whiskers represent 1.5× interquartile ranges, and scattered points represent outliers. Statistical significance is annotated as follows: Ns, not significant, *p < 0.05; **p < 0.01; ***p < 0.001; ****p < 0.0001.

Next, we analysed the relationship between the HMR subtypes and chemotherapy sensitivity (Figure 2E). Using the CTRPv2 and PRISM databases, we explored the response of the four HMR subtypes to common chemotherapeutic agents, including four compounds from CTRPv2 and nine compounds from PRISM. Lower AUC values indicated increased sensitivity to drug therapy. Compared to the other three subtypes, HMR‐C1 patients were insensitive to common adjuvant chemotherapeutic agents, showing the lowest sensitivity to paclitaxel and significantly higher resistance to oxaliplatin, cisplatin, fluorouracil, and capecitabine (*p* values: HMR‐C1 vs. HMR‐C2, C3, C4). Interestingly, HMR‐C3 patients exhibited significantly higher sensitivity to fluorouracil, mitomycin C, cisplatin, oxaliplatin, and capecitabine than the other subtypes, which may be associated with a favourable prognosis. In conclusion, the results suggest that the expression patterns of histone modification regulators are significantly associated with patient prognosis and can help guide drug selection, potentially providing new insights for the classification system of GC.

Together, these results identify four distinct HMR clusters (HMR‐C1 to C4) with significant differences in clinical outcomes and treatment responses and established HMR‐C1 as an independent poor prognostic factor for gastric cancer.

### 3.3 Biological Functional Features and Immune Landscape Under the Four Histone Modification Patterns

To further characterise the biological features of the four clusters, we analysed the biological functions and immune landscape differences among the HMR clusters. GSVA enrichment analysis of the “Hallmark” gene set revealed distant characteristics of HMR‐C1 (Figure 3A–B). HMR‐C1 was enriched in stromal/mesenchymal phenotype‐related pathways, with the highest activation level of Epithelial‐mesenchymal transition (EMT)—related pathways. Hypoxia and TGF‐β signalling were significantly more pronounced in HMR‐C1. Moreover, HMR‐C1 cells exhibited enrichment of pro‐tumour pathways, such as angiogenesis, and higher scores for cell proliferation‐related pathways (e.g., mitotic spindle). Furthermore, the IL2‐STAT5 signalling pathway displayed greater activity in HMR‐C1 compared to the other clusters. Enrichment of these pathways suggests the formation of a pro‐tumour inflammatory microenvironment in HMR‐C1 patients. HMR‐C2 was enriched in pathways such as MYC targets v1, G2M checkpoint, DNA repair, and Unfolded protein response. In addition, metabolic pathways, including bile acid metabolism and xenobiotic metabolism, showed significantly higher scores in HMR‐C3 patients, suggesting that metabolic inhibition may be a potential treatment strategy for HMR‐C3 patients.

**FIGURE 3:**
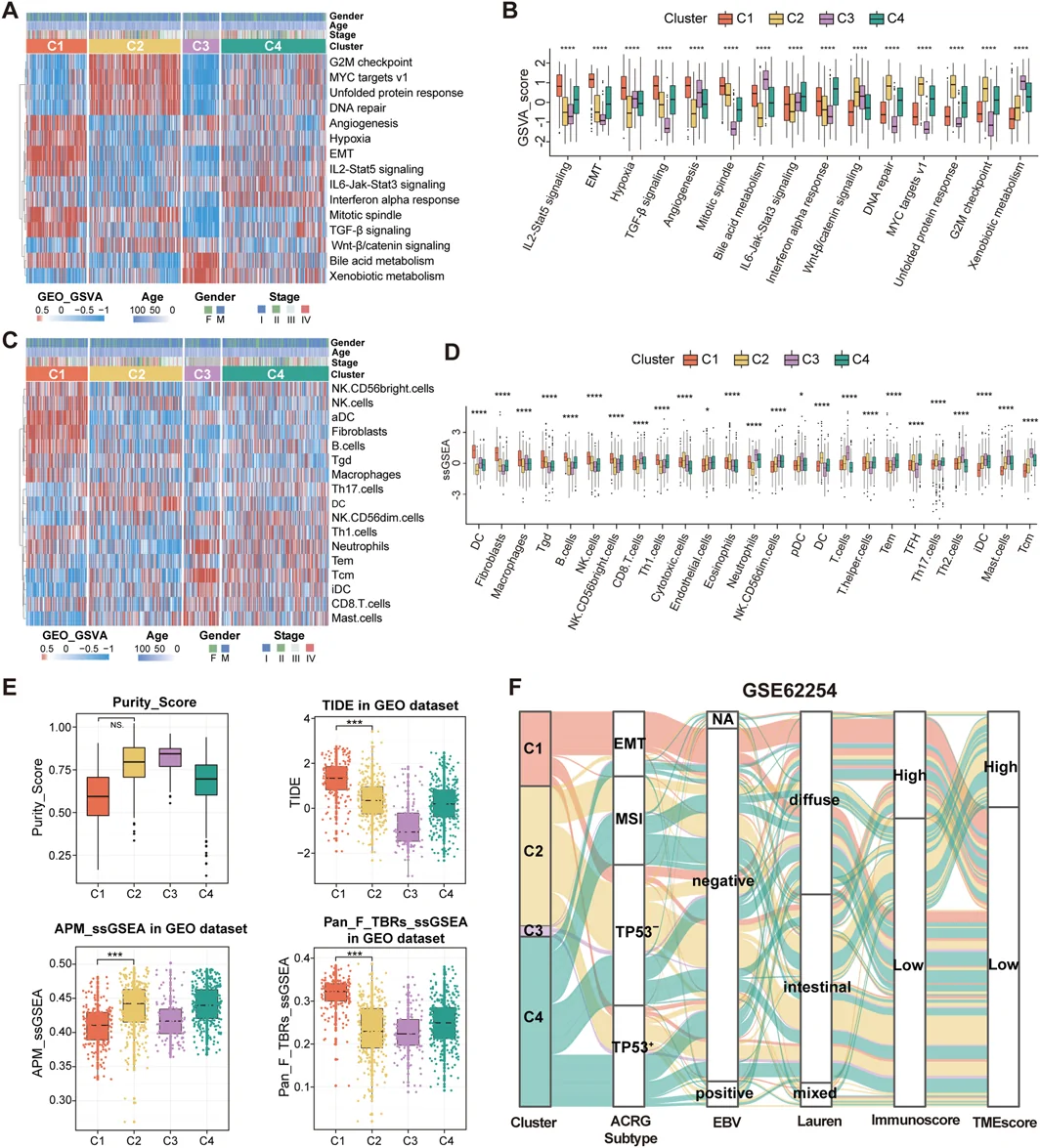
Biological functional features and immune landscape under the four histone modification patterns. (A) Heatmaps shows the GSVA results based on the “hallmark gene sets” in the four histone modification patterns in the meta‐GEO cohort. Red represents the activated pathways, and blue represents the inhibited pathways. Histone modification patterns are used as a sample annotation. (B) Boxplot of the GSVA results for the four histone modification patterns in the meta‐GEO cohort. Boxes represent interquartile ranges (25%–75% data range), horizontal lines in boxes represent medians, whiskers represent 1.5× interquartile ranges, and scattered points represent outliers. Statistical significance is annotated as follows: Ns, not significant, *p < 0.05; **p < 0.01; ***p < 0.001; ****p < 0.0001. (C) Heatmaps are generated to depict the ssGSEA immune cell infiltration analysis in the four histone modification patterns in the meta‐GEO cohort. The annotations are performed based on histone modification patterns and clinical information. (D) Boxplot of immune cell infiltration analysis by ssGSEA of the four histone modification patterns in the meta‐GEO cohort. Boxes represent 25%–75% of the values, the lines in the box represent the median, the whiskers represent 1.5 interquartile ranges, and the black dots represent outliers. (E) Boxplots showing the tumour purity of the four histone modification patterns in the meta‐GEO cohort, predicted TIDE values, and immune microenvironment scores calculated from the IOBR package. Boxes represent interquartile ranges (25%–75% data range), horizontal lines in boxes represent medians, whiskers represent 1.5× interquartile ranges, and scattered points represent outliers. (F) Sankey plot illustrates the association between the histone modification regulators clusters and different molecular subtypes in the meta‐GEO cohort.

Next, we performed ssGSEA on the meta‐GEO cohort to determine the relative infiltration abundance of immune cells in the TME across the four clusters (Figure 3C–D). The results showed that, compared with the other three clusters, HMR‐C1 exhibited unique TME characteristics, with increased infiltration of immune and stromal cells (enriched in fibroblasts), including activated dendritic cells (aDCs), fibroblasts, macrophages, Tgd cells, B cells, and NK cells. Evidence of innate immune activation was observed, reflected by the enrichment of Tgd cells, NK cells, macrophages, and aDCs, indicating that HMR‐C1 patients display TME features resembling the immune rejection phenotype. This phenotype is characterised by abundant immune cell infiltration in the stroma surrounding the tumour nest, where they are impeded by collagen fibres secreted by cancer‐associated fibroblasts rather than infiltrating the tumour core [31]. HMR‐C4 patients exhibited TME characteristics similar to the immune desert phenotype, characterised by immunosuppression and minimal specific immune cell infiltration. HMR‐C2 and HMR‐C3 displayed moderate levels of immune cell infiltration. The HMR‐C2 cluster showed abundant infiltration of DC and Th17 cells, while aDC and neutrophils were downregulated, suggesting potential inhibition of antigen recognition. In HMR‐C3 cells, T cell infiltration was the highest, with relatively abundant Th2 cells, central memory T cells (Tcm), and neutrophils.

Further analyses revealed that HMR‐C1 patients have low tumour purity (ESTIMATE score) [32] and a high predictive value for TIDE prediction score [21] (Figure 3E). A higher TIDE score indicates a greater likelihood of immune escape by tumour cells and a diminished response to immune checkpoint inhibitor (ICI) treatment. Furthermore, using the IOBR package [23], we calculated pan‐fibroblast TGFβ response signature (Pan‐F‐TBRS) and APM scores to assess different histone modification patterns. The results showed that HMR‐C1 patients exhibited the lowest APM scores and the highest Pan‐F‐TBRS scores (Figure 3E). These findings further support the association of the HMR‐C1 cluster with the immune exclusion phenotype: compared with other groups, HMR‐C1 patients have high levels of stromal components, abundant immune cell infiltration, and limited response to ICIs.

We further explored the microenvironmental features of GC within the GSE62254 cohort by examining the clinical characteristics reported in the literature and their correlation with the expression patterns of histone modification regulators [33, 34]. The Sankey diagram revealed that most HMR‐C1 patients were concentrated in the high‐immunoscore group, exhibiting a low TME score and an EMT phenotype (Figure 3F). Conversely, the HMR‐C3 subtype was mainly concentrated in the low‐immunoscore group of the IRS fraction. Additionally, this subtype displayed high tumour purity, a low TME score phenotype, and a high TIDE score. These findings indicate that histone modification patterns possess distinct activation states and biological pathway characteristics.

### 3.4 Construction and Validation of HR_score Model

Since patients in the HMR‐C1 group had the worst prognosis and were insensitive to adjuvant chemotherapy, we hypothesised that developing a scoring model that independently quantifies the histone modification status to identify HMR‐C1 patients may have potential clinical value (Figure 4A). To this end, we performed differential expression analysis between the HMR‐C1 and non‐HMR‐C1 groups in the meta‐GEO cohort and identified a total of 8020 DEGs (Supporting Information S2: Table 4‐S4), of which 4832 were downregulated and 3188 were upregulated in HMR‐C1 (screening criteria: *p* < 0.001). We further applied the Boruta algorithm and LASSO‐COX regression analysis to finally screen 10 genes (*ACVR1*, *BRD3*, *C11orf95*, *CRYAB*, *FERMT2*, *IFNA2*, *RAE1*, *UCKL1*, *VPS72*, *YTHDF1*) to construct histone modification regulator features and calculate the scores (Supporting Information S2: Table 5,6‐S4). This score was named HR_score (Figure 4A–B). In the meta‐GEO cohort, HMR‐C1 showed the highest median HR_score (Figure 4C). Using the surv_cutpoint function of the survminer package, we determined the optimal cutoff value for HR_score to classify patients into high‐ and low‐HR_score groups, and analysed the gene expression patterns of the histone modification regulators after dimensionality reduction by the Boruta algorithm (Figure 4D).

**FIGURE 4:**
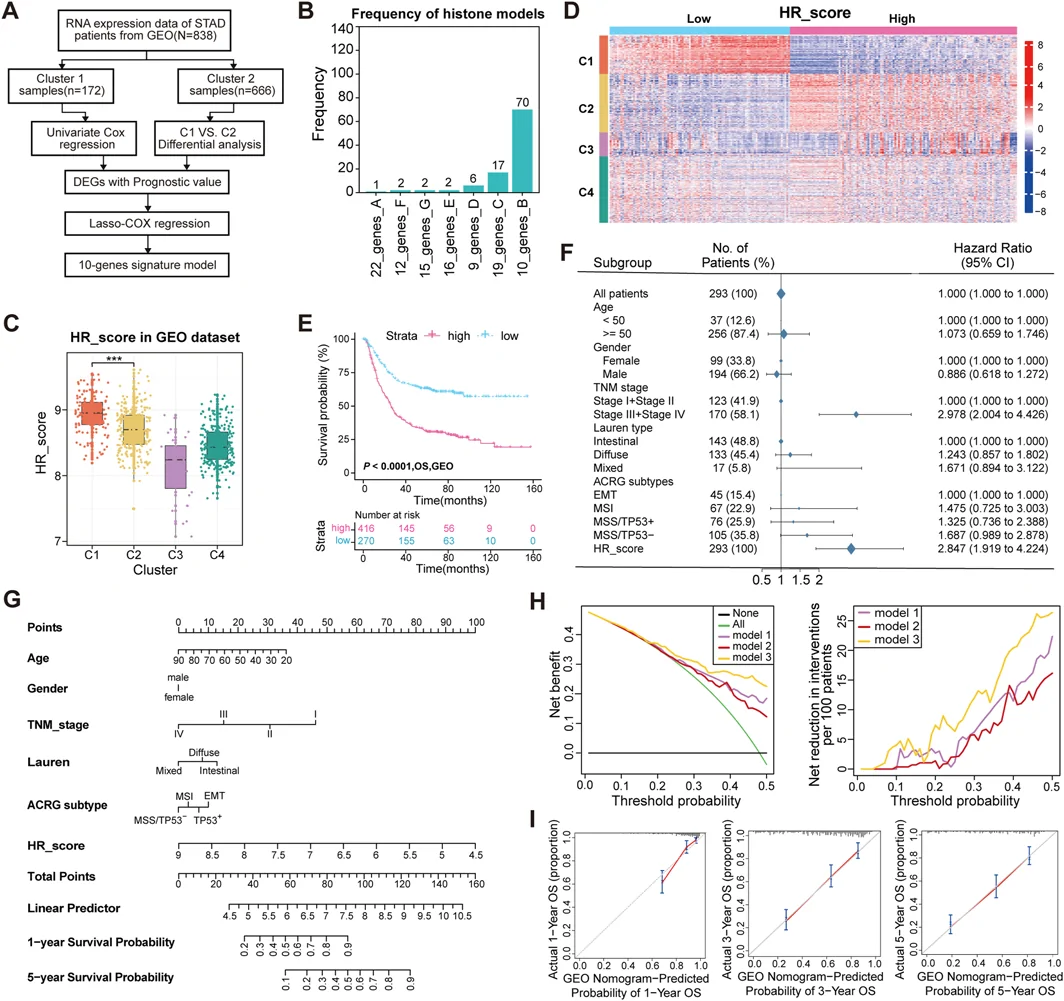
Construction and clinical validation of the HR_score. (A) Flowchart representing the establishment of model scores for histone modification regulators. (B) Bar graph showing the highest number of frequencies corresponding to the selection of the 10‐gene model in the LASSO regression analysis. (C) Box plot showing the distribution of HR_score across the four HMR subtypes in the meta‐GEO cohort. Statistical significance was determined using the Kruskal‐Wallis test. (D) Heatmap showing the gene expression patterns of histone modification regulator subtypes after dimensionality reduction by the Boruta algorithm. (E) Kaplan‐Meier survival curves demonstrating a significant difference in overall survival between the high‐ and low‐HR_score groups in the meta‐GEO cohort. *p*‐values were calculated using the two‐sided log‐rank test. (F) Forest plot showing a multifactorial COX risk regression model based on the GSE62254 cohort with patient gender, age, Lauren type, TNM stage, HR_score and ACRG subtype as covariates confirming HR_score as an independent prognostic marker. (G) Nomogram presenting the results of the multifactorial Cox regression model in a visual graphic. (H) Decision Curve Analysis (DCA) decision curve showing benefit scores on the vertical axis and probability thresholds on the horizontal axis. We plotted the clinical decision curves for model1 (clinical staging: TNM stage + Lauren type + ACRG subtype), model2 (HR_score), and model3 (clinical staging combined with HR_score), showing that HR_score provides better net benefit than “all” or “none”. (I) Calibration curves at 1, 3, and 5 years comparing the agreement between actual and predicted incidence rates were used to evaluate the validity of the multifactor cox regression model. Statistical significance is annotated as follows: ***p < 0.001.

To validate the expression of the 10 HR_score‐related genes in gastric cancer cells, we performed qRT‐PCR to examine their mRNA expression levels in the gastric cancer cell lines AGS and MKN45, using the normal gastric epithelial cell line GES‐1 as a control (Supporting Information S1: Figure S3). The results showed that, compared with GES‐1 cells, *VPS72*, *ACVR1*, *RAE1*, *CRYAB*, *YTHDF1*, *FERMT2*, *UCKL1*, *BRD3*, and *C11orf95* were significantly upregulated in both AGS and MKN45 cells (with positive coefficients in the HR_score formula; Table S2), whereas *IFNA2* expression was significantly downregulated (with a negative coefficient in the HR_score formula; Table S2). These trends were consistent with the bioinformatics analysis results.

We further investigated the prognostic impact and predictive value of the HR_score in patients with GC. In the meta‐GEO cohort, the high HR_score group had significantly shorter overall survival (OS, HR: 2.462, 95% CI: 1.952–3.106, *p* < 0.0001) (Figure 4E). These findings were validated in the TCGA‐STAD cohort, where the survival disadvantage of the high HR_score group remained statistically significant (Supporting Information S1: Figure S4A, OS, HR: 1.708, 95% CI: 1.170–2.494, *p* = 0.005). Consistent with the results of the meta‐GEO and TCGA‐STAD databases, in the external gastric cancer cohort TIMES001, Kaplan‐Meier curves showed that the high HR_score group had significantly shorter progression‐free survival (PFS, *p* = 0.039) (Supporting Information S1: Figure S4B). Multifactorial Cox regression models were employed to the GSE62254 and TCGA‐STAD cohorts, considering sex, age, TNM stage, Lauren subtype, and ACRG subtype as covariates. The results confirmed that the HR_score was an independent poor prognostic biomarker for assessing patient outcomes (meta‐GEO: HR 2.847, 95% CI 1.919–4.224, Figure 4F; TCGA‐STAD: HR: 1.627, 95% CI: 1.160–2.284, Supporting Information S1: Figure S4C).

Subsequently, we constructed nomograms using multivariate Cox regression based on the clinical characteristics of the GSE62254 and TCGA‐STAD cohorts. These nomograms visually presented the results of the Cox regression model and predicted 1‐year and 5‐year OS probabilities of GC patients (Figure 4G; Supporting Information S1: Figure S4D). To assess the clinical value of the line graphs, we plotted clinical decision curves for model 1 (clinical staging: TNM staging + Lauren's staging + molecular staging), model 2 (HR_score), and model 3 (clinical staging combined with HR_score), respectively. The curves demonstrated that the HR_score provided a more accurate answer compared to using “all” or “none” of the features, and model 3 (clinical features combined with HR_score) showed the highest value (Figure 4H). Furthermore, calibration curves were plotted to compare the predicted 1‐, 3‐, and 5‐year OS probabilities with the actual observations, demonstrating good agreement (Figure 4I; Supporting Information S1: Figure S4E). In summary, the above findings establish the 10‐gene HR_score as a robust, independent prognostic biomarker. It effectively identifies high‐risk HMR‐C1 patients and significantly enhances the predictive accuracy of clinical nomograms for gastric cancer survival across multiple validation cohorts.

### 3.5 Biological Functions and TME Characteristics of High and Low HR_score Groups

To further explore the characteristics of patients in the high and low HR_score groups, we conducted biological function and TME analyses. The results demonstrated that, similar to the HMR‐C1 subtype, patients in the high HR_score group exhibited activation of pathways related to cell growth, proliferation, and differentiation, such as EMT, mitotic spindle, Notch signalling, Hedgehog signalling, and TGF‐β signalling (Figure 5A–B; Supporting Information S1: Figure S5A, B). Additionally, the high HR_score group showed enrichment of immune cells, including fibroblasts, B cells, γδ T cells, and NK.CD56bright cells (Figure 5C–F), which may be associated with the immune exclusion phenotype. Moreover, analysis of the meta‐GEO cohort revealed that the high‐scoring group was insensitive to the most common adjuvant chemotherapeutic agents (Supporting Information S1: Figure S6A). These findings were consistent with results obtained from the TCGA‐STAD cohort (Supporting Information S1: Figure S6B).

**FIGURE 5:**
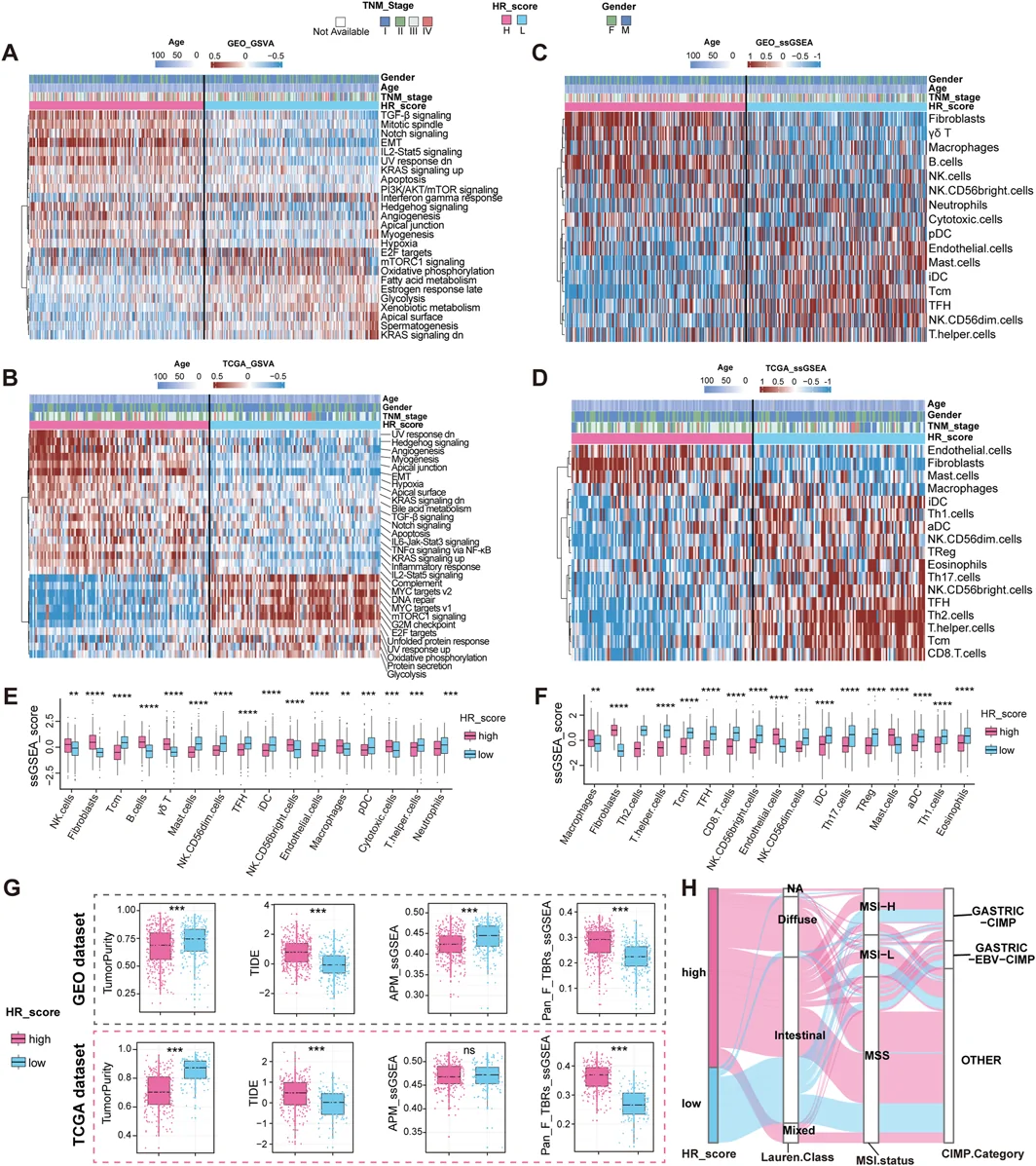
Biological characterization of histone modification regulator expression patterns in gastric cancer. (A, B) Heatmaps show the GSVA results based on the “hallmark gene sets” in the high and low HR_score groups in the meta‐GEO (A) and TCGA‐STAD (B) cohorts. Red represents activated pathways, and blue represents inhibited pathways. HR_score groups and clinical information are used as annotations. (C, D) Heatmaps depict the ssGSEA immune cell infiltration analysis within the high and low HR_score groups in the meta‐GEO (C) and TCGA‐STAD (D) cohorts. Annotations are performed based on the high and low HR_score groups and clinical information. (E, F) Boxplots of ssGSEA‐based immune cell infiltration analysis for the high and low HR_score groups in the meta‐GEO (E) and TCGA‐STAD (F) cohorts, respectively. Boxes represent interquartile ranges (25%–75% data range), horizontal lines in boxes represent medians, whiskers represent 1.5× interquartile ranges, and scattered points represent outliers. (G) Boxplots showing the graded tumour purity in the meta‐GEO cohort and TCGA‐STAD cohorts, the predicted TIDE values, and the immune microenvironment feature values calculated by the IOBR package. Boxes represent interquartile ranges (25%–75% data range), horizontal lines in boxes represent medians, whiskers represent 1.5× interquartile ranges, and scattered points represent outliers. (H) Sankey plot illustrates the association between the HR_score groups and other subtypes in the meta‐GEO cohort. Statistical significance is annotated as follows: Ns, not significant, *p < 0.05; **p < 0.01; ***p < 0.001; ****p < 0.0001.

Further analyses of both the meta‐GEO and TCGA‐STAD cohorts revealed that patients in the high HR_score group exhibited low tumour purity (ESTIMATE score) [31] and high predicted tumour immune dysfunction and exclusion values (TIDE prediction score) [20] (Figure 5G). This suggests that tumour tissues from high HR_score patients contain more non‐tumour components (e.g., stromal and immune cells) and were less responsive to immune checkpoint inhibitor therapy compared with low HR_score patients. We also calculated the immune microenvironment characteristics using the IOBR package [23] and analysed their distribution patterns in histone modification. Consistent with our previous findings, patients in the high HR_score group showed high Pan‐F‐TBRs values (Figure 5G), indicating a suppressed immune microenvironment. In contrast, patients in the low HR_score group from the meta‐GEO cohort exhibited high APM values, further confirming the activation of the antigen recognition function.

Moreover, we explored the relationship between HR_score and other subtypes in the TCGA‐STAD cohort (Figure 5H). Our analysis revealed an association between HR_score and Lauren subtype, CIMP classification, and MSI status. Most patients with low HR_score had the diffuse Lauren subtype. In contrast, approximately two‐thirds of patients with high HR_score had MSI status, suggesting a potential link to worse immunotherapy outcomes.

In conclusion, the above findings confirm that HR_score can effectively identify patients with the HMR‐C1 subtype. Patients in the high HR_score group exhibit features of oncogenic pathway activation, immune exclusion microenvironment, chemotherapy resistance, and enrichment of MSI status, further supporting HR_score as a potential biomarker for prognosis prediction and individualised treatment guidance in gastric cancer.

### 3.6 Multi‐Omic Analysis

To further explore the prognostic correlation between the HR_score and GC, we initially investigated the differences in somatic mutations between the high and low HR_score groups. The top 20 genes with the highest mutation frequencies in the high and low HR_score groups included *ACVR2A*, *RNF43*, *TTK*, *MBD6*, *ESRP1*, etc (Figure 6A). However, no significant differences in mutation frequencies were observed between the two groups.

**FIGURE 6:**
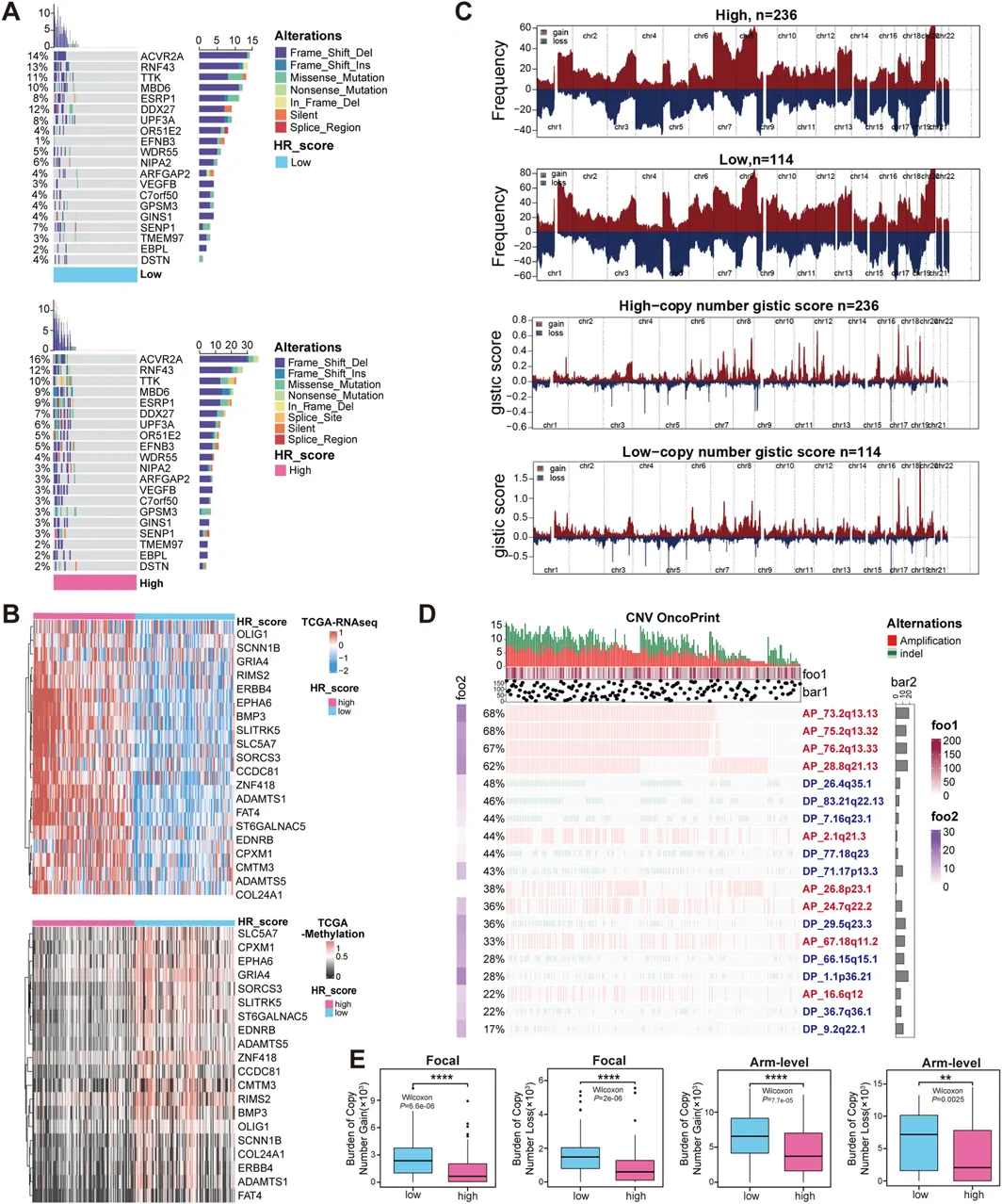
Copy number profiles and epigenetic alterations of histone modification patterns in gastric cancer. (A) Waterfall plot shows somatic mutation frequency in the high HR_score group (top), and the low HR_score group (bottom). Each column in the graph represents one patient. The top bar indicates the overall cellular mutation frequency of the patient. The numbers on the left show the mutation frequency for each gene. The bars on the right show the proportion of each mutation type. (B) Dual‐track heatmap showing the correlation between HR_score and the molecular landscape; the upper panel represents transcriptomic expression and the lower panel represents DNA methylation levels. (C) Copy number profiles of histone modification patterns in gastric cancer demonstrate the GISTIC score, and gain/loss frequency of the high and low scoring groups. (D) Oncoplot showing the most frequent altered (amplification or deletion) of copy number events sorted according to frequency, with red representing Amplification, green representing indel, each column representing a sample, and each row representing a chromosomal locus. (E) Quantitative analysis of focal‐level and arm‐level copy number variation burdens. Boxes represent the interquartile range, and horizontal lines indicate the median. Statistical significance is annotated as follows: Ns, not significant, *p < 0.05, **p < 0.01, ***p < 0.001, ****p < 0.0001.

Changes in DNA methylation are considered useful biomarkers for diagnosis, prognosis, and response to treatment. Hypomethylation may promote immune evasion *i* in tumour cells with high mutational and high copy number burdens. Therefore, we conducted a differential methylation analysis between the high and low HR_score subgroups using TCGA‐STAD methylation data. Simultaneously, we analysed the TCGA‐STAD RNA‐seq data to screen for DEGs. Our screening process yielded 20 genes (*p* < 0.001 and log2FC ≥ 1) that exhibited both hypomethylation and high expression levels in the high‐scoring group (Figure 6B). Previous studies have shown that *ERBB4* is frequently amplified in GC and is associated with advanced disease stages [35] and poor prognosis [36], and its mutations may contribute to GC development [37]. Meanwhile, ST6GALNAC5 may mediate breast cancer brain metastasis by enhancing tumour cell adhesion to brain endothelial cells and facilitating passage through the blood‐brain barrier [38]. The heatmap showed a positive correlation between the transcriptome expression levels of these 20 genes and HR_score, while methylation levels were negatively correlated with HR_score (Figure 6B).

Furthermore, we assessed the overall condition of copy number alterations, including amplification and deletion, in the high and low HR_score subgroups using the GISTIC2.0 algorithm [39]. The low HR_score group exhibited higher frequencies of both copy number gains and losses (Figure 6C). We also identified the chromosomal loci most frequently affected by copy number events (amplification or deletion) (Figure 6D). The identified amplification loci were: 73.2q13.13, 73.2q13.32, 76.2q13.33, 28.8q21.13, 2.1q21.3, 26.8p23.1, 24.7q22.2, 67.18q11.2, and 16.6q12. The deletion sites included: 26.4q35.1, 83.21q22.13, 7.16q23.1, 77.18q23, 71.17p13.3, 29.5q23.3, 66.15q15.1, 1.1p36.21, 36.7q36.1, and 9.2q22.1. Moreover, the low‐score group exhibited significantly higher burdens of focal and arm‐level gains and losses (Figure 6E), indicating a higher copy number variation burden compared with the high‐score group. This phenomenon may be associated with the dual‐edged effect of genomic instability [40]. Collectively, these results may help explain the prognostic differences between the high and low HR_score groups.

### 3.7 Identify the Potential Therapeutic Agents in the Different HR_Score Groups

To explore the implications of the HR_score for GC treatment, we utilised the CMap method described in the Method section [41] (Supporting Information S2: Table 7‐S4) to predict the potential compounds that would most likely be the chemotherapy choice for GC patients. Our analysis identified MS.275 and 4,5‐dianilinophthalimide as the drugs most likely to be effective for the high and low HR_score groups, respectively (Figure 7A). Additionally, the correlation matrix map revealed the top 20 small‐molecule compounds enriched in both the high‐ and low‐score groups, including MK.886, STOCK1N.35874, and STOCK1N.35696 (Figure 7B). These findings offer valuable insights into the clinical treatment of GC.

**FIGURE 7:**
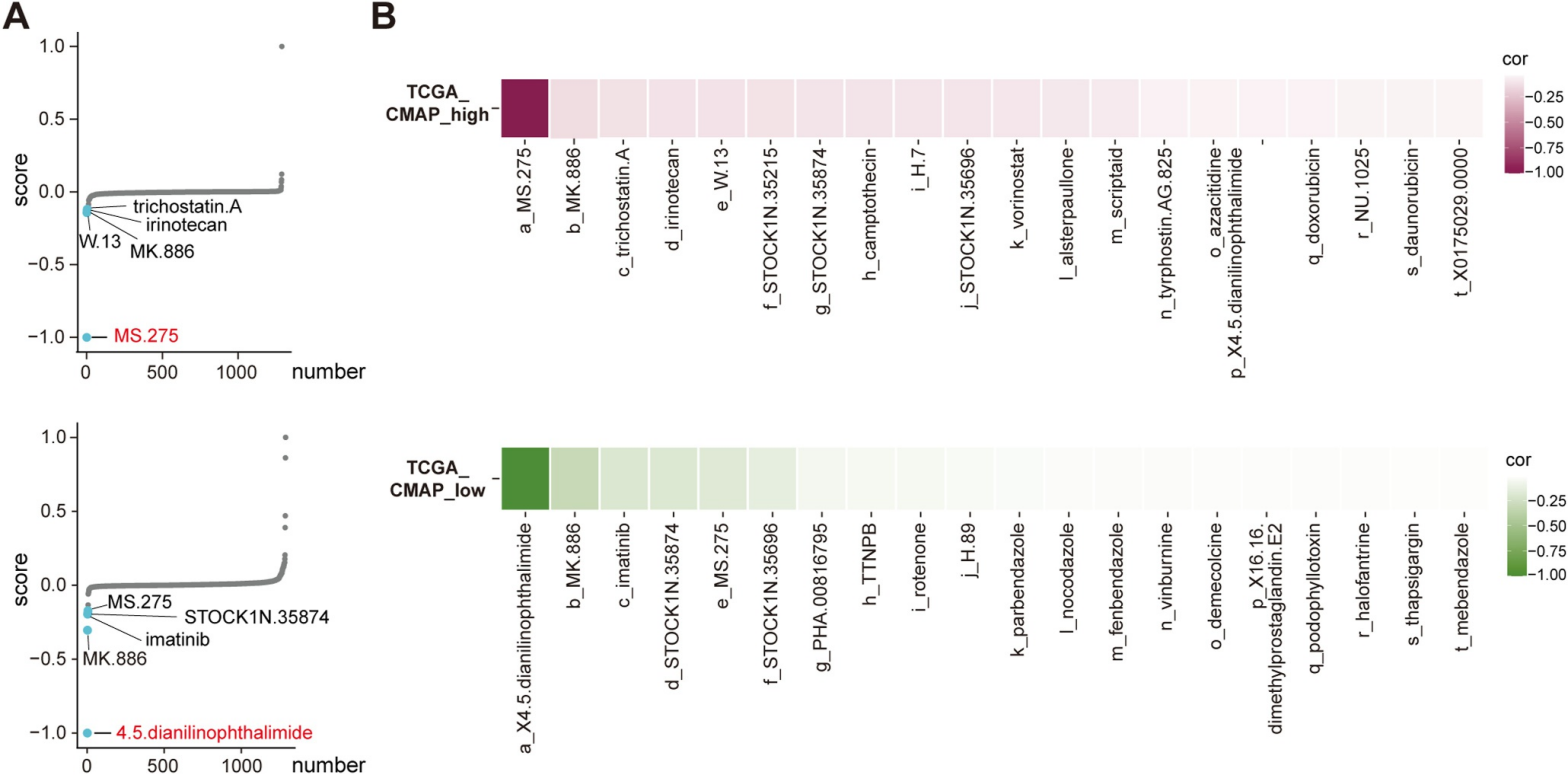
Prediction of possible therapeutic drugs for high and low HR_score groups. (A) Small molecule drugs that may have potential were obtained by CMap analysis comparing the high and low score groups with normal tissues in the TCGA‐STAD cohort, and the results suggested that MS.275, 4.5‐dianilinophthalimide were the most likely drugs for treating the high HR_score group versus the low HR_score group in gastric cancer, respectively. (B) Correlation matrix plot demonstrating the top 20 small molecule compounds for each of the negative connectivity scores in the high and low HR_score groups.

## 4 Discussion

In this study, we utilised consensus clustering and analysed the transcriptome data of 76 histone acetylation and methylation regulators and identified four distinct expression patterns: HMR‐C1, HMR‐C2, HMR‐C3, and HMR‐C4. Among these, HMR‐C1 was significantly associated with poor prognosis and limited efficacy of adjuvant chemotherapy and immunotherapy. Subsequently, we employed LASSO‐based regression and multivariate stepwise Cox regression analysis to screen 10 genes and constructed a quantitative scoring model, HR_score, which enables quantification of histone modification status and the differentiation of the HMR‐C1 subtypes.

We found that the genes included in the HR_score calculation are closely related to tumour occurrence and development. As a subunit of the histone acetyltransferase complex, *VPS72* can promote hepatocellular carcinoma progression and interact with MYC/ACTL6A to enhance oncogene transcription [42]. *ACVR1* is a type I receptor of the BMP/TGF‐β family, which is highly expressed in gastric cancer and upregulated by Helicobacter pylori infection, promoting tumour proliferation, migration, and invasion [43, 44]. *RAE1* drives the migration and invasion of gastric cancer cells through the ERK/MAPK pathway, and its high expression is closely associated with poor prognosis [45, 46]. *CRYAB* is a heat shock chaperone protein, and its overexpression can induce tumour formation, inhibit apoptosis, and exert an oncogenic effect [47]. *YTHDF1* is a key reader protein of m6A modification, involved in gastric cancer progression by regulating translation and mRNA stability, and is associated with chemotherapy and immunotherapy resistance [48]. In addition, *FERMT2*, *UCKL1*, *BRD3*, and *C11orf95* mediate the formation of an immunosuppressive microenvironment by promoting tumour metastasis and proliferation [49, 50, 51, 52]. Notably, *IFNA2* is the only gene with a negative regression coefficient in the model; it encodes type I interferon, which has broad‐spectrum anti‐tumour immune activity [53]. Collectively, these 10 genes interact at multiple levels, supporting HR_score as a potential tool for prognostic prediction and individualised treatment guidance in gastric cancer.

HR_score is an independent poor prognostic factor for gastric cancer, with patients in the high HR_score group exhibiting the worst prognosis, while HMR‐C1 showed the highest median HR_score. Similar to the HMR‐C1 subtype, patients in the high HR_score group exhibited significant activation of EMT, cancer stemness, and pathways related to malignant cancer phenotypes. The Notch [54], TGF‐β [55], and Hedgehog [56] pathways, as core tumour regulatory pathways, can promote tumour cell proliferation, invasion, metastasis, and immune escape upon abnormal activation, ultimately mediating poor prognosis. Additionally, patients in the HMR‐C1 and high HR_score groups presented a phenotype of high stromal components and abundant immune cell infiltration, which is highly consistent with the immune exclusion phenotype. Notably, both groups had high TIDE scores, suggesting poor response to ICI therapy, a conclusion further supported by their low APM and high Pan‐F‐TBRs features. The TGF‐β signal enriched in Pan‐F‐TBRs can drive tumour immune escape by promoting the formation of an immune exclusion phenotype [57, 58], while defects in antigen presentation further weaken immunotherapy efficacy [59]. The above pathway activation and immune microenvironment abnormalities together constitute an important molecular basis for tumour progression and poor prognosis in patients with HMR‐C1 and high HR_score. These patients are insensitive to common adjuvant chemotherapy drugs and have the worst prognosis, suggesting that adjuvant chemotherapy alone is unlikely to provide significant benefit to this population. Therefore, constructing a scoring model that can accurately identify patients with the HMR‐C1 subtype holds important clinical translational value.

Remarkably, R‐loops, as key products of transcription‐replication conflicts, have been established as a major source of genomic instability, including copy number variations [60]. In this study, patients in the low HR_score group exhibited a higher CNV burden, suggesting that tumors in this group may harbour elevated R‐loop accumulation or impaired R‐loop resolution. First, aberrantly accumulated RNA‐DNA hybrids in the cytoplasm can activate innate immune pathways such as cGAS‐STING [61, 62], thereby inducing anti‐tumour immune responses and promoting the infiltration of immune cells, including T cells and neutrophils (Figure 5). Meanwhile, high levels of CNV are often accompanied by extensive DNA damage and genomic rearrangements, which can generate abundant tumour neoantigens that are subsequently recognised by the immune system, eliciting anti‐tumour immune responses [63]. Additionally, unresolved R‐loop‐mediated DNA damage can activate apoptotic pathways, which may also limit the malignant proliferation potential of cells with high CNV burden [64]. Collectively, these factors may explain why the low HR_score group exhibits a favourable prognosis despite having a high CNV burden. This hypothesis warrants further experimental validation in future studies.

To explore the therapeutic implications of the HR_score further, we employed the CMap methodology to predict potential therapeutic agents. MS.275 and 4,5‐dianilinophthalimide emerged as the most promising candidates for the high and low HR_score groups, respectively. MS‐275 is a potent, orally available, and selective class I histone deacetylase inhibitor that induces autophagy and apoptosis in tumour cells. It has demonstrated antiproliferative activity in vitro in various human cancer cell lines, including breast, colon, and lung cancers [65]. MS‐275 has also shown synergistic antitumour effects when combined with cisplatin or IL‐2 in oesophageal squamous or renal cell carcinoma [66, 67]. In contrast, 4,5‐dianilinophthalimide, a selective inhibitor of the epidermal growth factor receptor signalling pathway, has potent in vivo antitumour activity against different cancer types, including bladder metastatic cell carcinoma [68, 69]. The CMap results can guide new disease‐directed drug development and provide therapeutic options for GC.

These findings suggest that HR_score holds potential for several clinical applications. It can serve as a screening tool to identify gastric cancer patients who are unlikely to benefit from adjuvant chemotherapy alone, thereby avoiding unnecessary toxicity. Additionally, given the strong association between high HR_score and immune exclusion, HR_score may help predict poor response to immune checkpoint inhibitor therapy, thereby guiding more rational patient selection for immunotherapy. Furthermore, HR_score may assist in identifying patients who could benefit from therapeutic strategies such as histone deacetylase inhibitors, helping clinicians develop more precise treatment strategies for individual patients.

Although our study proposes a molecular subtype based on histone modification regulators in gastric cancer, several limitations exist. First, we only analysed histone acetylation and methylation regulators. These two types of modifications are currently the most in‐depth studied histone modification types, and their roles in tumorigenesis, development, and clinical prognosis are the clearest. Future studies should incorporate other atypical histone modification types, such as lactylation [70], crotonylation [71], propionylation [72], butyrylation [72], and hydroxybutyrylation [73], to further improve prognostic models for gastric cancer patients. In addition, the external validation cohort TIMES001 used in this study is from a single centre, with patients predominantly microsatellite‐stable (accounting for 91.9%); the sample size of this cohort is relatively small (*n* = 86), and the patient characteristics may have limitations in regionality and specific disease subtypes. Therefore, the universality and clinical utility of HR_score need to be further validated in prospective, multi‐center cohorts with larger sample sizes and broader coverage of molecular subtypes. Future prospective studies are warranted to evaluate the performance of HR_score in guiding adjuvant chemotherapy and immunotherapy decisions in real‐world clinical settings. Moreover, further in vitro and in vivo experimental studies are necessary to elucidate the underlying mechanisms of these genes in gastric cancer progression and to strengthen the biological evidence supporting the clinical utility of HR_score.

## 5 Conclusion

In conclusion, this study comprehensively evaluated biological functions, TME cell infiltration, and molecular features associated with the expression patterns of histone modification regulators. It demonstrated how epigenetic mechanisms generate and enhance tumour heterogeneity and influence TME characteristics, leading to a poor prognosis. The study also suggested potentially effective therapeutic interventions. Moreover, the study introduced the HR_score, an evaluation tool with clinical translational value. This tool accurately identifies patients with the HMR‐C1 subtype by quantifying histone modification status and predicting patient prognosis and adjuvant chemotherapy response. The HR_score may provide more precise and comprehensive therapeutic guidelines for patients with GC.

## Author Contributions

**Yue Huang:** conceptualization, formal analysis, methodology, writing – original draft, visualization. **Ya Gao:** conceptualization, formal analysis, methodology, writing – original draft, visualization. **Fuli Xie:** data curation, investigation, software, visualization. **Xiaoxiang Rong:** conceptualization, funding acquisition, project administration, writing – review and editing. **Rui Zhou:** conceptualization, funding acquisition, methodology, writing – review and editing. **Zhenhua Huang:** conceptualization, writing – review and editing. **Kuncai Liu:** investigation, software. **Jiayun Guan:** investigation, software. **Zewei Li:** software. **Siting Zheng:** methodology, writing – review and editing. **Jianhua Wu:** project administration, supervision. **Guangting He:** formal analysis, funding acquisition, methodology, project administration. **Yanhua Ou:** project administration, visualization, writing – review and editing. **Huiying Sun:** formal analysis, project administration, supervision, writing – original draft, writing – review and editing.

## Funding

This work was supported by the National Natural Science Foundation of China (Grant 82073375 to Xiaoxiang Rong; Grant 82102731 to Rui Zhou), Guangdong Basic and Applied Basic Research Foundation (Grant 2025A1515012516 to Xiaoxiang Rong; Grant 2025A1515012253 to Rui Zhou), Scientific and Technological Planning Project of Guangzhou City (Grant 2023A04J2331 to Guangting He).

## Conflicts of Interest

The authors declare no conflicts of interest.

## Supporting information


Supporting information S1

Supporting Information S2

**Table S1:** Summary of the gastric cancer cohorts used in this study.

**Table S2:** Multivariate cox regression coefficients of the 10 included genes in HR_score calculation.

**Table S3:** Primers for RT‐qPCR.

## Data Availability

The public data used in this study are available at: GSE62254 (http://www.ncbi.nlm.nih.gov/geo/query/acc.cgi?acc = GSE62254); GSE57303 (http://www.ncbi.nlm.nih.gov/geo/query/acc.cgi?acc = GSE57303); GSE29272 (http://www.ncbi.nlm.nih.gov/geo/query/acc.cgi?acc = GSE29272); GSE15459 (http://www.ncbi.nlm.nih.gov/geo/query/acc.cgi?acc = GSE15459); and TCGA‐STAD (https://xenabrowser.net/datapages/?cohort = TCGA Stomach Cancer (STAD)). Methylation data for TCGA‐STAD used in this study are also available from UCSC Xena (https://xenabrowser.net). Original bulk RNA‐seq data of the TIMES001 cohort are available at GSA‐Human (https://ngdc.cncb.ac.cn/gsa‐human/, accession number: HRA005161).
