# Supplementary material for: Expression Patterns of Histone Modification Regulators Predict Prognosis and Microenvironment Characteristics in Gastric Cancer
Source: IET Syst Biol. 2026 Nov 4;20(1):e70089. doi: 10.1049/syb2.70089 (PMC13734686; doi:10.1049/syb2.70089)
Supplement: Supplementary file 1 [file SYB2-20-e70089-s002.pdf]

Figure S1. Principal Component Analysis visualization of sample distribution before and after batch effect correction.

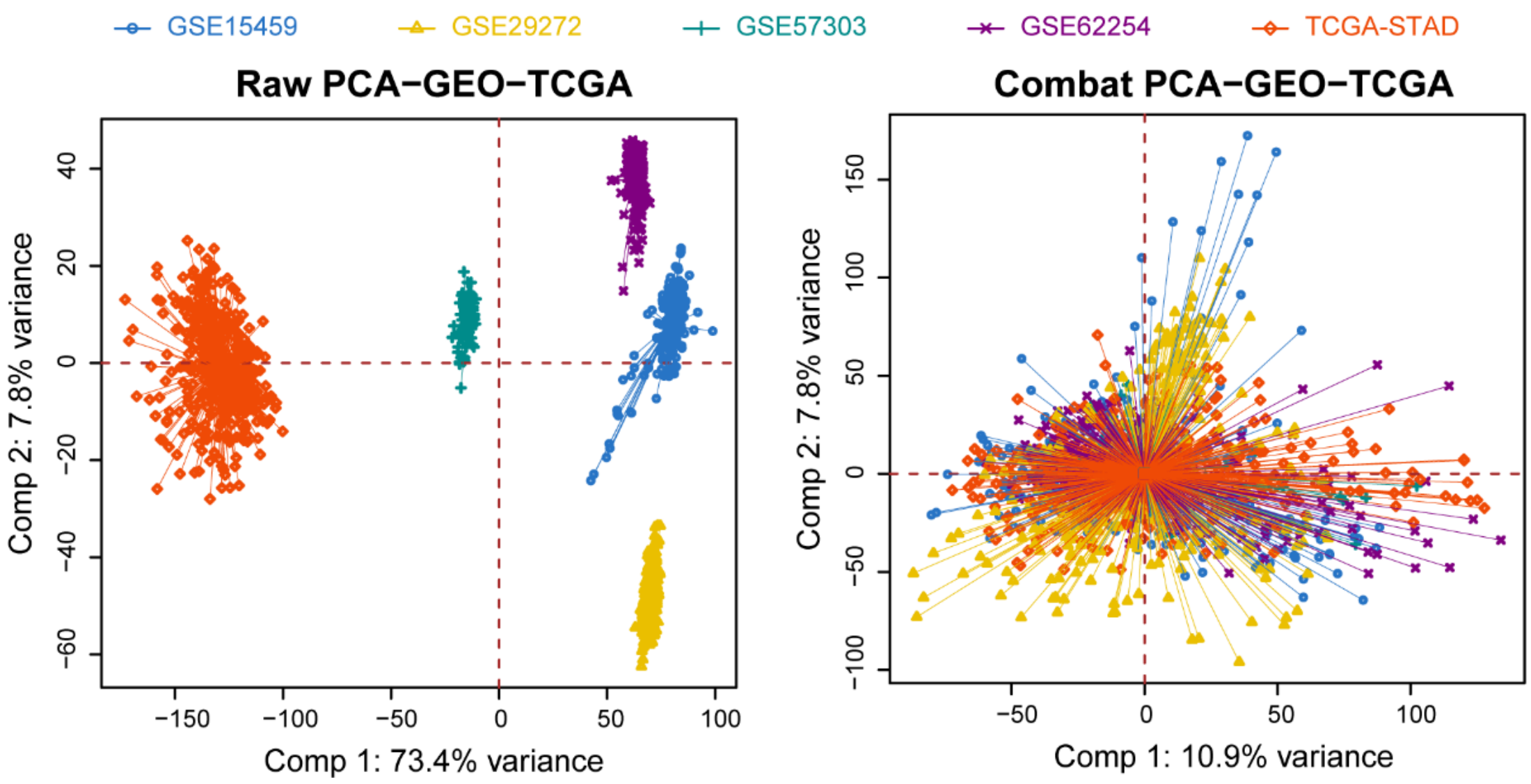

This figure shows the distribution of samples from each dataset (GSE62254, GSE57303, GSE29272, GSE15459, and TCGA-STAD) in the principal component space before and after batch effect correction. The left panel (Raw) shows the PCA results of samples without batch effect correction, and the right panel shows the PCA results of samples corrected using the ComBat algorithm. After correction, the samples from different cohorts became well-mixed, indicating effective removal of batch effects.

Figure S2. Consensus clustering of histone modification regulators.

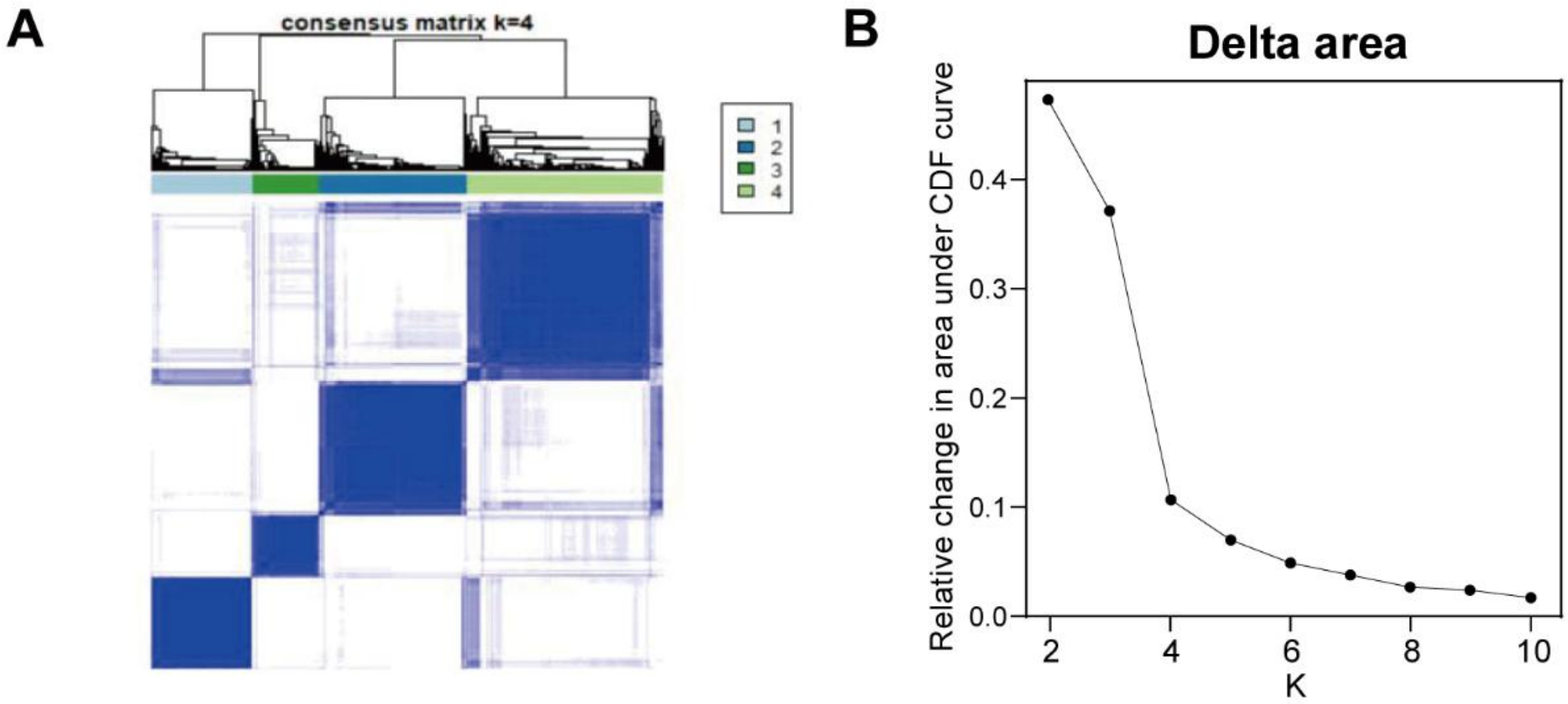

A. Clustering matrix plot of optimal histone modification typing in gastric cancer patients, indicating the differentiation of the samples into 4 subtypes by unsupervised consistent clustering method (k=4).

B. Delta Area Plot: This plot shows the relative change in the area under the CDF curve for k and k-1 compared to the CDF curve. In this paper, k=4 is taken.

Figure S3. mRNA expression levels of the 10 HR_score-related genes in gastric cancer cell lines.

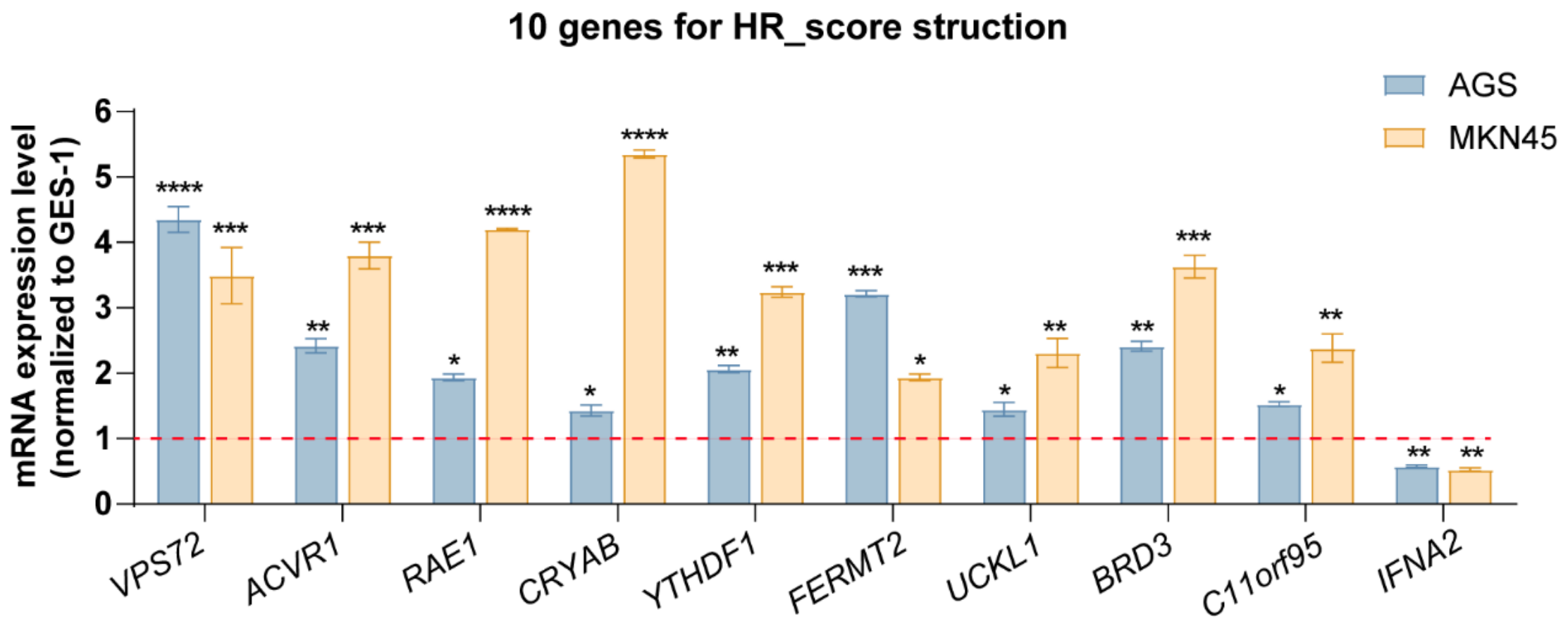

The expression levels of *ACVR1*, *BRD3*, *C11orf95*, *CRYAB*, *FERMT2*, *IFNA2*, *RAE1*, *UCKL1*, *VPS72*, and *YTHDF1* were examined by quantitative real-time PCR in AGS and MKN45 gastric cancer cells, with GES-1 normal gastric epithelial cells as the control. The relative mRNA expression was normalized to GES-1 (set as 1). *β-ACTIN* was used as an internal control. Data are presented as mean ± SD of three independent experiments. Statistical significance was determined by Student's t test (**P* < 0.05, ***P* < 0.01, ****P* < 0.001, *****P* < 0.0001).

Figure S4. Validation of histone modification scores.

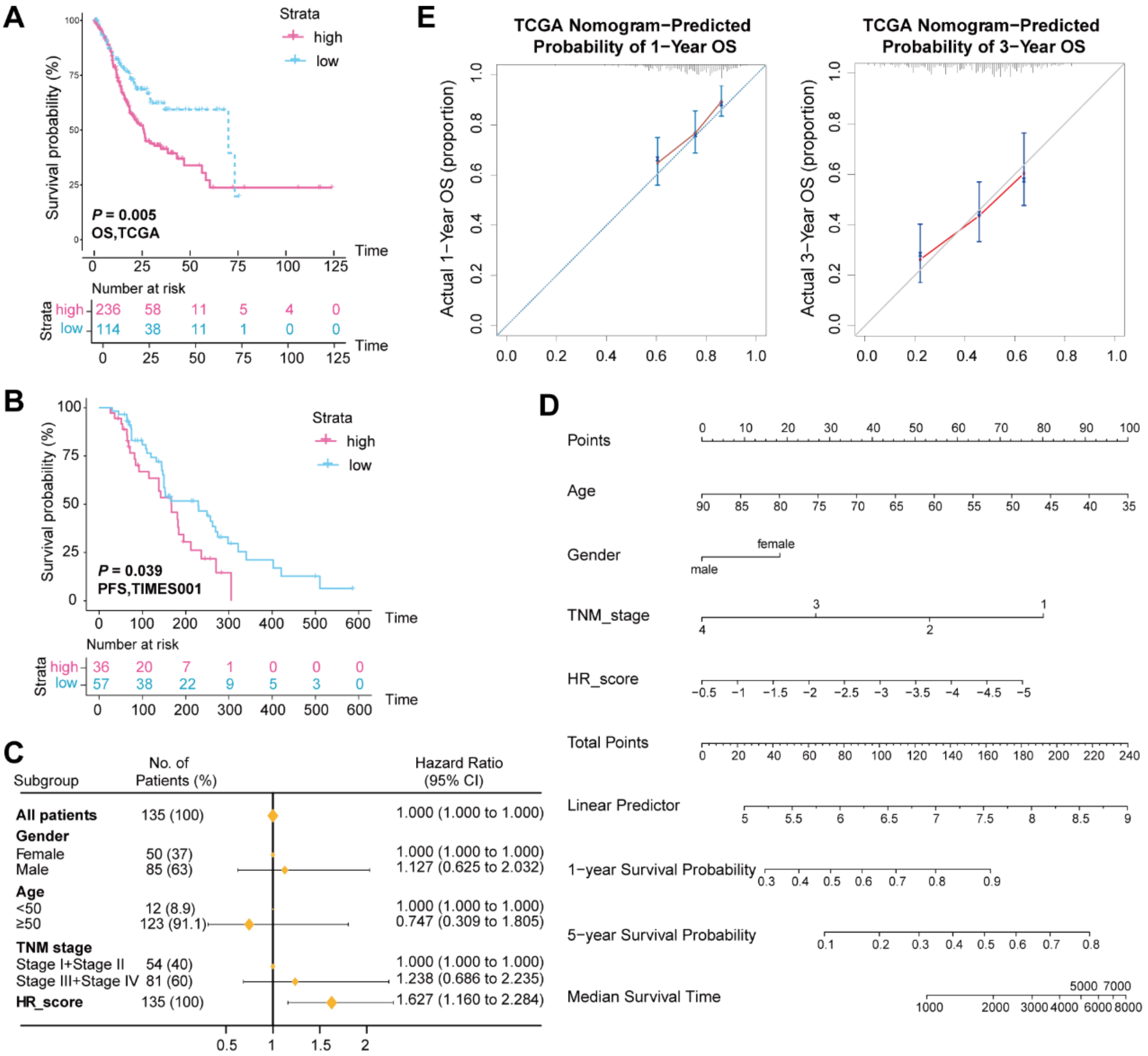

A. Kaplan-Meier curves of overall survival (OS) in the TCGA-STAD cohort based on histone modification regulator patterns.

B. Kaplan-Meier curves of progression-Free Survival (PFS) in the TIMES001 cohort based on histone modification regulator patterns.

C. Forest plot shows that a multivariate COX risk regression model based on the TCGA-STAD cohort with patient sex, age, Lanren classification, TNM stage, and ACRG typing as a covariate confirmed HR_score as an independent prognostic marker.

D. Nomogram presents the results of the multivariate Cox regression model are presented as visual figures.

E. A calibration curve showing a scatter plot of actual and predicted incidence to evaluate the multivariate cox regression model.

Figure S5. GSVA enrichment scores of hallmark gene sets in high and low HR_score groups in the meta-GEO and TCGA-STAD cohorts.

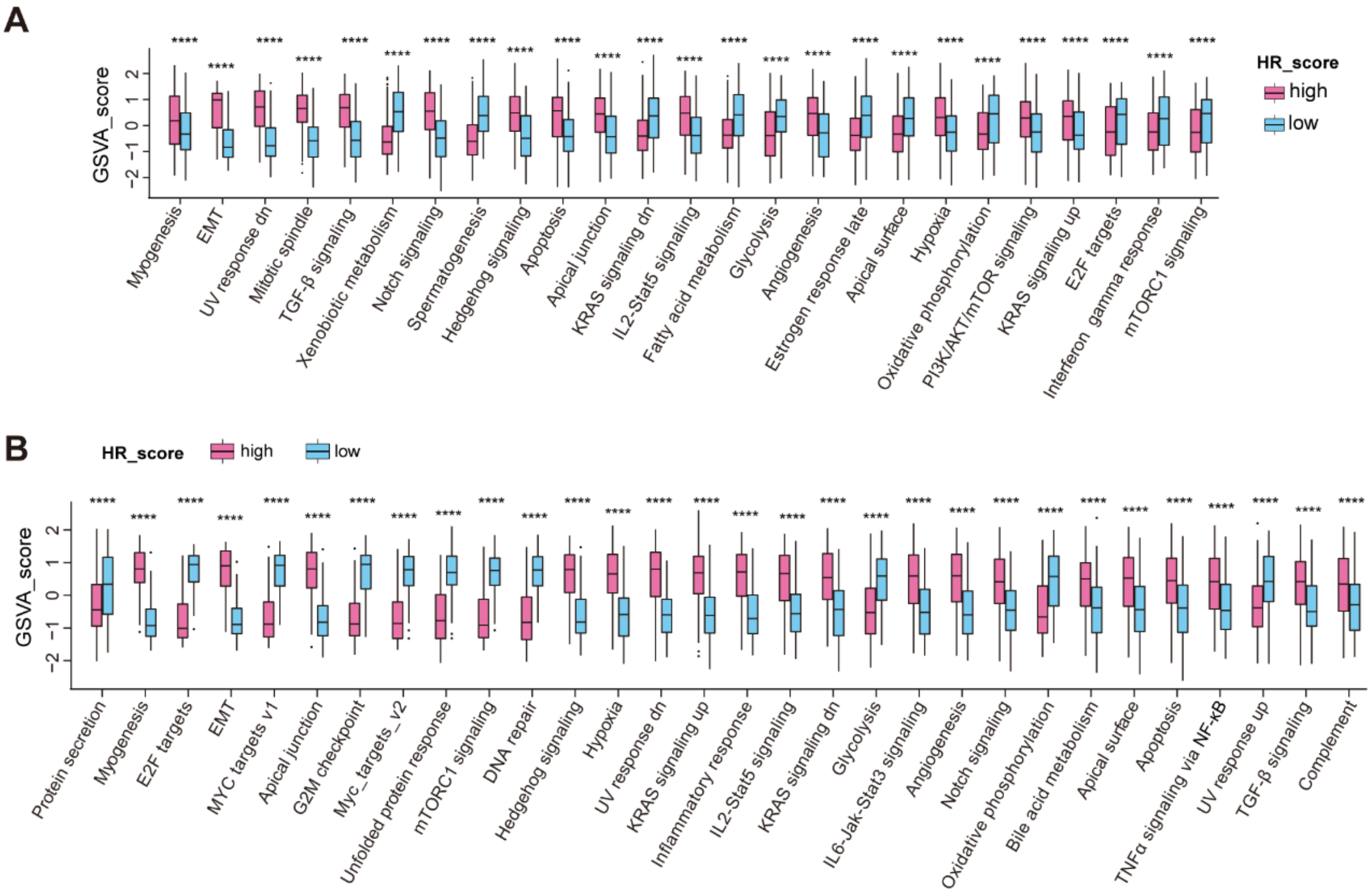

A. Boxplots of GSVA results for the high and low HR_score groups in the meta-GEO cohort.

B. Boxplots of GSVA results for the high and low HR_score groups in the TCGA-STAD cohort. Boxes represent interquartile ranges (25%~75% data range), horizontal lines in boxes represent medians, whiskers represent 1.5x interquartile ranges, and scattered points represent outliers. ****P < 0.0001**.**

Figure S6. Sensitivity of high and low HR_score groups to commonly used chemotherapeutic agents.

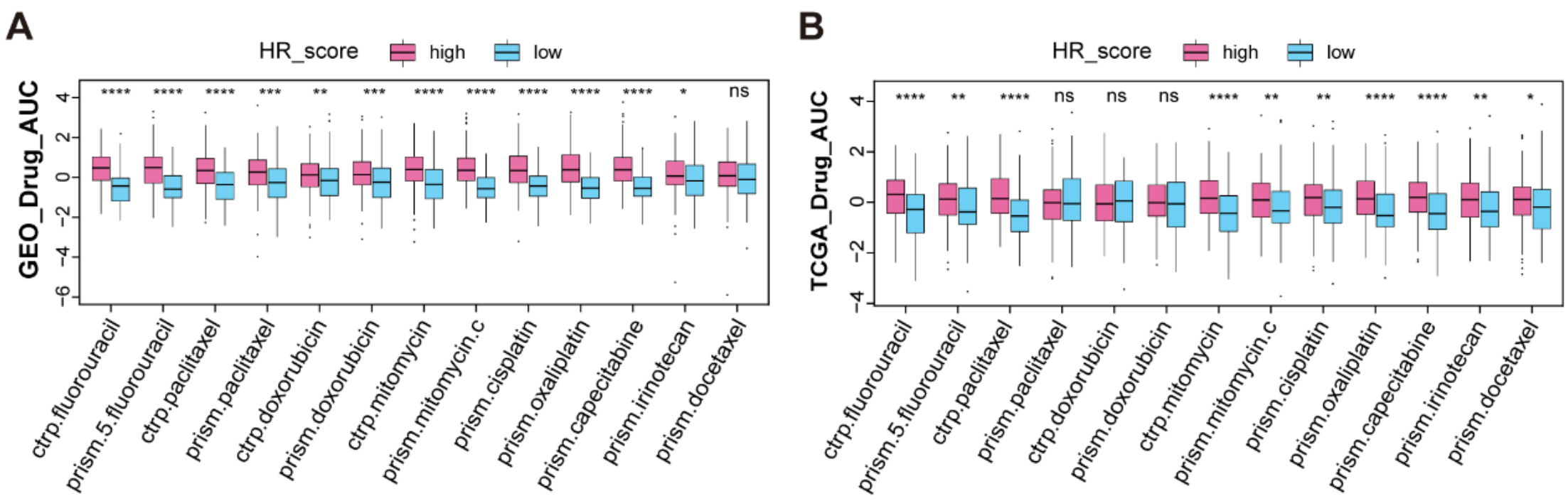

A-B. Box plots showing the sensitivity of the meta-GEO cohort **(A)** and the TCGA-STAD cohort **(B)** to commonly used chemotherapeutic agents, grouped by high and low scores. Statistical significance is annotated as follows: ns, not significant; *P < 0.05; **P < 0.01; ***P < 0.001; ****P < 0.0001.
