# Supplementary material for: Expression Patterns of Histone Modification Regulators Predict Prognosis and Microenvironment Characteristics in Gastric Cancer
Source: IET Syst Biol. 2026 Nov 4;20(1):e70089. doi: 10.1049/syb2.70089 (PMC13734686; doi:10.1049/syb2.70089)
Supplement: Supplementary file 2 [file SYB2-20-e70089-s001.docx]

**S2 Table.** Multivariate Cox regression coefficients of the 10 included genes in HR_score calculation.

| Gene | β |
| --- | --- |
| ACVR1 | 0.2092996 |
| BRD3 | 0.0553844 |
| C11orf95 | 0.0451483 |
| CRYAB | 0.1431185 |
| FERMT2 | 0.1166078 |
| IFNA2 | -0.212207 |
| RAE1 | 0.1449949 |
| UCKL1 | 0.0837885 |
| VPS72 | 0.3193562 |
| YTHDF1 | 0.1388062 |
