# Supplementary material for: Expression Patterns of Histone Modification Regulators Predict Prognosis and Microenvironment Characteristics in Gastric Cancer
Source: IET Syst Biol. 2026 Nov 4;20(1):e70089. doi: 10.1049/syb2.70089 (PMC13734686; doi:10.1049/syb2.70089)
Supplement: Supplementary file 3 [file SYB2-20-e70089-s004.docx]

**S3 Table. Related to Figure S3.** Real-time quantity PCR primers sequences.

Abbreviations: h, human.

| Gene |  | Sequence (5’-3’) |
| --- | --- | --- |
| h-ACTB | F  R | CATGTACGTTGCTATCCAGGC  CTCCTTAATGTCACGCACGAT |
| h-VPS72 | F  R | GCTCTGACAGTCGGAAGTCTA  GTTCCTCCTGGGTTAGTGGC |
| h-ACVR1 | F  R | GTGAAGGTCTCTCCTGCGGTA  GCCATCGTTGATGCTCAGTGA |
| h-RAE1 | F  R | GCAAAGTGTTTACGGCATCGT  GGGTATATCACGTCAGCACAGTA |
| h-CRYAB | F  R | AGGTGTTGGGAGATGTGATTGA  GGATGAAGTAATGGTGAGAGGGT |
| h-YTHDF1 | F  R | ACCTGTCCAGCTATTACCCG  TGGTGAGGTATGGAATCGGAG |
| h-FERMT2 | F  R | TCTGACCATGCTCTCTGGTG  AGTTCTTCGGGGTGTCTGATA |
| h-UCKL1 | F  R | GGCCGCACACAACAACTTC  GGGCACCTTGACACTCTTCC |
| h-BRD3 | F  R | TCAAATTGAACCTGCCGGATT  TGCATACATTCGCTTGCACTC |
| h-C11orf95 | F  R | CTCAAGGTGAGCACCATCAAG  GCTCCTCAGGCGTGAAGTC |
| h-IFNA2 | F  R | GCTTGGGATGAGACCCTCCTA  CCCACCCCCTGTATCACAC |
