# Supplementary material for: Expression Patterns of Histone Modification Regulators Predict Prognosis and Microenvironment Characteristics in Gastric Cancer
Source: IET Syst Biol. 2026 Nov 4;20(1):e70089. doi: 10.1049/syb2.70089 (PMC13734686; doi:10.1049/syb2.70089)
Supplement: Supplementary file 4 [file SYB2-20-e70089-s005.docx]

**S1 Table.** Summary of the gastric cancer cohorts used in this study. The datasets GSE62254, GSE57303, GSE29272, and GSE15459 were merged into the meta-GEO cohort (n = 838).

| Datasets | Platform | Country | No. of patients | Survival |
| --- | --- | --- | --- | --- |
| GSE62254 | GPL570 | USA | 300 | OS |
| GSE57303 | GPL570 | China | 70 | OS |
| GSE29272 | GPL96 | USA | 268 | OS |
| GSE15459 | GPL570 | Switzerland | 200 | OS |
| TCGA-STAD | RNA-seq | USA | 407 | OS |
| TIMES001 | RNA-seq | China | 86 | PFS |
